# Synthesis, Molecular Properties Estimations, and Dual Dopamine D_2_ and D_3_ Receptor Activities of Benzothiazole-Based Ligands

**DOI:** 10.3389/fchem.2017.00064

**Published:** 2017-09-12

**Authors:** Moritz Schübler, Bassem Sadek, Tim Kottke, Lilia Weizel, Holger Stark

**Affiliations:** ^1^Institute of Pharmaceutical Chemistry, Goethe University Frankfurt Frankfurt, Germany; ^2^Department of Pharmacology and Therapeutics, College of Medicine and Health Sciences, United Arab Emirates University Al Ain, United Arab Emirates; ^3^Institute of Pharmaceutical and Medicinal Chemistry, Heinrich Heine Universität Düsseldorf Duesseldorf, Germany

**Keywords:** benzothiazoles, dopamine D_2S_/D_3_ receptor, *in vitro* activities, privileged structures, drug-likeness

## Abstract

Neurleptic drugs, e.g., aripiprazole, targeting the dopamine D_2S_ and D_3_ receptors (D_2S_R and D_3_R) in the central nervous system are widely used in the treatment of several psychotic and neurodegenerative diseases. Therefore, a new series of benzothiazole-based ligands (**3-20**) was synthesized by applying the bioisosteric approach derived from the selective D_3_Rs ligand **BP-897** (**1**) and its structurally related benz[*d*]imidazole derivative (**2**). Herein, introduction of the benzothiazole moiety was well tolerated by D_2S_R and D_3_R binding sites leading to antagonist affinities in the low nanomolar concentration range at both receptor subtypes. However, all novel compounds showed lower antagonist affinity to D_3_R when compared to that of **1**. Further exploration of different substitution patterns at the benzothiazole heterocycle and the basic 4-phenylpiperazine resulted in the discovery of high dually acting D_2S_R and D_3_R ligands. Moreover, the methoxy substitution at 2-position of 4-phenylpiperazine resulted in significantly (22-fold) increased D_2S_R binding affinity as compared to the parent ligand **1**, and improved physicochemical and drug-likeness properties of ligands **3-11**. However, the latter structural modifications failed to improve the drug-able properties in ligands having un-substituted 4-phenylpiperazine analogs (**12-20**). Accordingly, compound **9** showed in addition to high dual affinity at the D_2S_R and D_3_R [*K*_i_ (*h*D_2S_R) = 2.8 ± 0.8 nM; *K*_i_ (*h*D_3_R) = 3.0 ± 1.6 nM], promising clogS, clogP, LE (*h*D_2S_R, *h*D_3_R), LipE (*h*D_2S_R, *h*D_3_R), and drug-likeness score values of −4.7, 4.2, (0.4, 0.4), (4.4, 4.3), and 0.7, respectively. Also, the deaminated analog **10** [*K*_i_ (*h*D_2S_R) = 3.2 ± 0.4 nM; *K*_i_ (*h*D_3_R) = 8.5 ± 2.2 nM] revealed clogS, clogP, LE (*h*D_2S_R, *h*D_3_R), LipE (*h*D_2S_R, *h*D_3_R) and drug-likeness score values of −4.7, 4.2, (0.4, 0.4), (3.9, 3.5), and 0.4, respectively. The results observed for the newly developed benzothiazole-based ligands **3-20** provide clues for the diversity in structure activity relationships (SARs) at the D_2S_R and D_3_R subtypes.

## Introduction

The dopaminergic systems in the central nervous system (CNS) have been comprehensively studied over the past five decades (Missale et al., 1998). Dopamine (DA) exerts its (patho)physiological effects through five distinct G-protein coupled receptors (D_1−5_ receptors), grouped in two classes, Gs-coupled D_1_-like (D_1_R and D_5_R) and Gi-coupled D_2_-like (D_2_R, D_3_R, and D_4_R), that vary in their signal transduction, binding shape and physiological effects (Missale et al., 1998). Moreover, an alternative splicing of D_2_R mRNA leads to generation of two isoforms: D_2_ short (D_2S_) and D_2_ long (D_2L_), which have been linked (though not completely) with presynaptic and postsynaptic populations of D_2_Rs, respectively (Joyce and Millan, 2005). Accordingly, it has been found that the D_2S_R is mainly considered as a presynaptic receptor, whereas, the D_2L_R as a postsynaptic receptor (Joyce and Millan, 2005), comparable to the D_3_R (Lader, 2008). However, it has been proposed that D_3_R, in addition to the classical postsynaptic location, is also localized in the presynapse, where it modifies release and synthesis of DA (Sokoloff et al., 1990; Noeske et al., 2006; Bocker et al., 2007; Boeckler and Gmeiner, 2007). Furthermore, it has been convincingly demonstrated that *h*D_2_R and *h*D_3_R are highly homologous (Stark, 1998), sharing 78% of sequence identity in the transmembrane domains (Kaufmann and Bückmann, 1941), including the binding site (Levey et al., 1993). Thus, this sequence identity has introduced difficulties in the design of ligands selectively targeting D_2s_R and/or D_3_R, since such ligands could offer more effective neuroleptic drugs with a favorable side effect profile in therapeutic management of central diseases, e.g., schizophrenia and Parkinson's disease. Accordingly, the newer atypical antipsychotic drugs, e.g., aripiprazole, with high affinity for D_2_Rs and D_3_Rs show a unique pharmacological profile at treating the positive symptoms of schizophrenia and have the potential to treat negative and cognitive symptoms, while also mitigating risk of weight gain and movement side effects (Swainston Harrison and Perry, 2004; Casey and Canal, 2017; Tuplin and Holahan, 2017).

**Figure 1:**
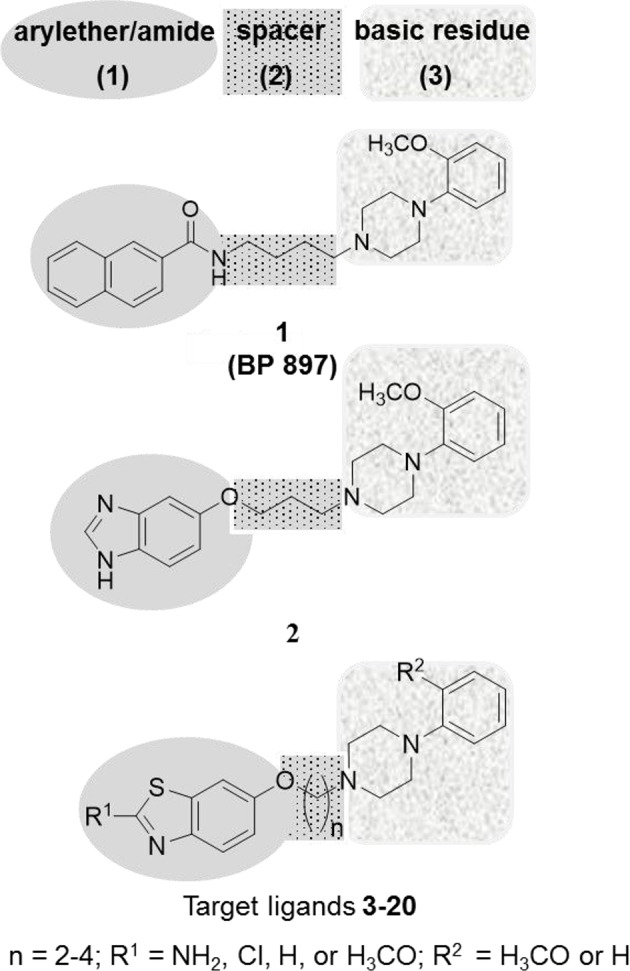
Features similarities between the lead structures **1**, **2** and target compounds (**3-20**).

In the past two decades, medicinal chemists have succeeded, by using ligand-based approaches, in developing selective D_2S_R, D_3_R, and dually acting D_2S_R/D_3_R ligands such as benz[*d*]imidazolyl ether **2** (Pilla et al., 1999; Garcia-Ladona and Cox, 2003; Boeckler and Gmeiner, 2007; Tomic et al., 2007; Moller et al., 2014; Stucchi et al., 2015). Interestingly, ligand **1** has earlier been described as a selective D_3_R ligand with a 66-fold higher selectivity over D_2S_R (Pilla et al., 1999). This former clinical candidate bears distinctive minimal pharmacophoric structure elements for many D_3_R ligands, which could be divided into three substructures: a lipophilic aryl moiety connected to an amide or ether (**1**), which is linked by a linear alkyl spacer (**2**) to a basic amine residue with aryl substitution (**3**) (Figure 1; Pilla et al., 1999; Garcia-Ladona and Cox, 2003). The basic 4-phenylpiperazine substructure (**3**) also described as “privileged structural surrogate” has proved to be essential for ligand interaction with selectivity for the D_2S_Rs and D_3_Rs (Hackling et al., 2003; Moller et al., 2014). Accordingly, ligand efficacy and selectivity between D_2S_R and D_3_R activation were found to be strongly influenced by regiochemistry and the nature of functional groups attached to the pyrazolo[1,5-a]pyridine heterocycle moiety. (Hackling et al., 2003; Moller et al., 2014). Also, the aliphatic tetramethylene spacer (**2**) in ligand **1** demonstrated the optimum distance between the two aromatic systems (**1**) and (**3**) (Hayes et al., 1992; Sokoloff et al., 1992; Noeske et al., 2006; Bocker et al., 2007; Boeckler and Gmeiner, 2007). Moreover, the amide function in numerous previously developed D_2S_R/D_3_R selective ligands, e.g., **1**, has been described as a weak H-bond acceptor and was directly connected to the aryl moiety (**1**) (Wright and Rosowsky, 2002; Hackling et al., 2003; Moller et al., 2014). Furthermore, the lipophilic residue in the aryl moiety (**1**) has convincingly been demonstrated to tolerate plenty of variations including biphenyl, heteroaryl or cycloalkyl substituents, resulting in many D_2_R/D_3_R ligands (Figure 1) (Murray et al., 1996; Bettinetti et al., 2002; Moller et al., 2014). Moreover, the recently described benz[*d*]imidazolyl ether (**2**) exhibited preference to the D_2S_Rs and D_3_Rs, respectively (Figure 1; Tomic et al., 2007; Moller et al., 2014). Keeping this in view and in continuation of our endeavors toward providing tools with which further exploration of the role of the dopaminergic system, the development of high-affinity D_2_R/D_3_R ligands was undertaken. It was envisaged that the heterocyclic appendage of benzothiazole, as a lipophilic bioisosterical replacement of aryl moiety (**1**), with the basic 4-phenylpiperazine aryl substitution (**3**) by an ether of varying alkyl spacer length (**2**) is worth the attempt. Accordingly, the replacement of the amide group present in ligand **1** with an ether structure was undertaken to investigate whether the affinity of the developed ligands will be modified toward balanced D_2S_R/D_3_R. In addition, the inclusion of different substituents at 2-position of benzothiazole (**1**) or 4-phenylpiperazine (**3**) was carried out to explore further the modulating role of hydrogen bound donors and lipophilic interaction with the heterocyclic π-system present in the novel ligands **3-20**. Moreover, all the title ligands **3-20** were subjected to Molinspiration Property, Osiris Property Explorer, and MolSoft toolkits to predictively assess their physicochemical properties. Furthermore, initial metric analyses were conducted to predictably quantify the lipophilicity (clogP), the water solubility (clogS), the ligand efficiency (LE), lipophilicity-dependent ligand efficiency (LELP), and lipophilic efficiency (LipE) important for verification of drug-likeness of the novel compounds (**3-20**) presented in the current study.

## Experimental procedures

### Chemistry

#### General remarks

All starting reagents/chemicals and dry solvents of commercial quality were purchased from the commercial suppliers VWR Darmstadt/Germany, Acros, Geel/Belgium, Alfar Aesar, Ward Hill/U.S.A., Sigma-Aldrich, St. Louis/USA, Fluka, München/Germany and Carl Roth, Karlsruhe/Germany. ^1^H-NMR and ^13^C-NMR spectra were recorded on a Bruker AMX 250 (250 MHz), a Bruker AMX 300 (300 MHz) or a Bruker AMX 400 (400 MHz) spectrometer (Bruker, Germany). ^1^H-NMR data are reported in the following order: chemical shift (**δ**) in ppm downfield from tetramethylsilane as internal reference; multiplicity (br, broad; s, singlet; d, doublet; t, triplet; q, quartet; quin, quintet; m, multiplet); approximate coupling constants (*J*) in Hertz (Hz). ESI-MS was performed on a Fisons Instruments VG Platform II (Manchester, Great Britain) in positive polarity. Data (m/z) are listed as mass number (M+H^+^) and relative intensity (%). MALDI_MS was performed on Voyager DE STR (Per Septive Biosystems). High Resolution Mass Spectrometry (HRMS) was carried out on a Thermo Scientific MALDI LTQ XL Orbitrap (Waltham, USA). Thin layer chromatography was performed on silica gel 60 F_254_ aluminum sheets (Merck, Germany). Preparative column chromatography was performed on silica gel 63–200 μM (Merck, Germany). Melting points were determined on a Büchi B510 melting point apparatus (Büchi, Switzerland).

#### Synthesis of new compounds 3-20 and their respective intermediates

##### 1-(2-bromoethoxy)-4-nitrobenzene (22a)

4-Nitrophenole (**21**, 3.0 g, 22 mmol) was diluted into 100 ml acetone. 1,4-dibromobutane (16.53 g, 88 mmol), anhydrous potassium carbonate (3.0 g, 22 mmol) and a catalytic amount of potassium iodide was added. The suspension was heated under reflux for 24 h. After cooling the yellow-white suspension was filtered. The yellow filtrate was evaporated to give a yellow oily raw product. The product was purified by filtration with petrolether (60–90°C) and dicloromethane over silicagel to obtain a pure yellow solid; yield 60%; C_8_H_8_BrNO_3_; ^1^H-NMR (CDCl_3_, 400 MHz) δ = 3.61 (t, *J* = 6.10 Hz, 2H, OCH_2_C*H*_2_Br), 4.32 (t, *J* = 6.11 Hz, 2H, OC*H*_2_CH_2_Br), 6.91 (d, *J* = 9.11 Hz, 2H, Ar-2-*H*, -6-*H*), 8.14 (d, *J* = 9.16 Hz, 2H, Ar-3-*H*, -5-*H*), ESI-MS (m/z): 246.0 (M-H^+^, 100), 247.4 (M-H^+^, 99), 249.0 (M-H^+^, 6).

##### 1-(3-bromopropoxy)-4-nitrobenzene (22b)

Intermediate **22b** was synthesized according to above described procedure for **22a**, however, with 1,3-dibromopropne (17.77 g, 88 mmol) to yield a white solid; yield: 86%; C_9_H_10_BrNO_3_; ^1^H-NMR (CDCl_3_, 300 MHz): δ = 2.29 (p, *J* = 6.02 Hz, 2H, OCH_2_C*H*_2_CH_2_Br) 3.54 (t, *J* = 6.30 Hz, 2H, OCH_2_ CH_2_C*H*_2_Br), 4.14 (t, *J* = 5.82 Hz, 2H, OC*H*_2_CH_2_CH_2_Br), 6.89 (d, *J* = 9.00 Hz, 2H, Ar-2-*H*, -6-*H*), 8.12 (d, *J* = 9.12 Hz, 2H, Ar-3-*H*, -5-*H*); ESI-MS (m/z): 234.9 (M-Br+NH_4_OH+Na^+^, 100), 236.0 (M-Br+NH_4_OH+Na^+^, 16).

##### 1-(4-bromobutoxy)-4-nitrobenzene (22c)

Intermediate **22c** was synthesized according to above described procedure for **1a**, however, with 1,4-dibromobutane (19.00 g, 88 mmol) affording a yellow oil; yield: 90%; C_10_H_12_BrNO_3_; ^1^H-NMR (CDCl_3_, 400 MHz): δ = 1.90–2.05 (m, 4H, OCH_2_C*H*_2_C*H*_2_CH_2_Br) 3.43 (t, *J* = 6.37 Hz, 2H, OCH_2_CH_2_CH_2_C*H*_2_Br), 4.03 (t, *J* = 5.78 Hz, 2H, OC*H*_2_CH_2_CH_2_CH_2_Br), 6.87 (d, *J* = 9.15 Hz, 2H, Ar-2-*H*, -6-*H*), 8.12 (d, *J* = 9.13 Hz, 2H, Ar-3-*H*, -5-*H*); ESI-MS (m/z): 297.9 (M-Na^+^, 100), 295.9 (M-Na^+^, 98), 296.7 (M-Na^+^, 10).

##### 4-(2-bromoethoxy)-aniline (23a)

(4.2 mmol) was dissolved with 50 ml ethyl acetate. 100 mg Pd/C (10% m/m) and a few drops glacial acetic acid were added. The suspension was stirred at room temperature in a Parr apparatus with a pressure of 5 bar hydrogen for 1 h. After filtration of the catalyst the yellow solution was evaporated to dryness to give a Yellow oil (0.76 g, yield: 100%); C_8_H_10_BrNO; ^1^H-NMR (CDCl_3_, 300 MHz), δ = 3.52 (t, *J* = 6.34 Hz, 2H, OCH_2_C*H*_2_Br), 4.14 (t, *J* = 6.34 Hz, 2H, OC*H*_2_CH_2_Br), 4.38 (s, br, 2H, Ar-N*H*_2_), 6.58 (d, *J* = 8.86 Hz, 2H, Ar-2-*H*, -6-*H*), 6.69 (d, *J* = 8.85 Hz, 2H, Ar-3-*H*, -5-*H*); ESI-MS (m/z): 215.6 (M-H^+^, 100), 216.6 (M-H^+^, 9), 217.7 (M-H^+^, 98).

##### 4-(3-bromopropoxy)aniline (23b)

Intermediate **23b** was synthesized according to above described procedure for **23a**, however, starting from **22b** (4.2 mmol). Brown oil; yield: 100%; C_9_H_12_BrNO; ^1^H-NMR (CDCl_3_, 300 MHz): δ = 2.19 (p, *J* = 6.17 Hz, 2H, OCH_2_C*H*_2_CH_2_Br) 3.52 (t, *J* = 6.49 Hz, 2H, OCH_2_ CH_2_C*H*_2_Br), 3.96 (t, *J* = 5.82 Hz, 2H, OC*H*_2_CH_2_CH_2_Br), 4.59 (s, br, 2H, ArN*H*_2_), 6.59 (d, *J* = 8.79 Hz, 2H, Ar-2-*H*, -6-*H*), 6.69 (d, *J* = 8.84 Hz, 2H, Ar-3-*H*, -5-*H*); ESI-MS (m/z): 229.8 (M-H^+^, 100), 230.7 (M-H^+^, 9), 231.7 (M-H^+^, 97).

##### 4-(4-bromobutoxy)aniline (23c)

Intermediate **23c** was synthesized according to above described procedure for **23a**, however, starting from **22c** (4.2 mmol). Yellow oil; yield: 100%; C_10_H_14_BrNO; ^1^H-NMR (CDCl_3_, 300 MHz) δ = 1.80–2.00 (m, 4H, OCH_2_C*H*_2_C*H*_2_CH_2_Br) 3.41 (t, *J* = 6.62 Hz, 2H, OCH_2_CH_2_CH_2_C*H*_2_Br), 3.86 (t, *J* = 6.03 Hz, 2H, OC*H*_2_CH_2_CH_2_CH_2_Br), 6.59 (d, *J* = 9.03 Hz, 2H, Ar-2-*H*, -6-*H*), 6.67 (d, *J* = 8.96 Hz, 2H, Ar-3-*H*, -5-*H*); ESI-MS (m/z): 243.7 (M-H^+^, 100), 245.7 (M-H^+^, 98).

##### 6-(2-bromoethoxy)-2-aminobenzothiazole (24a)

**23a** (4.4 mmol) and potassium thiocyanate (1.71 g, 17.6 mmol) were solved in 25 ml glacial acetic acid. The resultant brown solution was cooled on an ice bath. Dropwise bromine (0.71 g, 4.4 mmol) dissolved in 5 ml glacial acetic acid was added, while the temperature was held constant at 4°C. Afterwards the orange suspension was stirred at room temperature overnight. The resulting solid was filtered of and the yellow filtrate was concentrated in vacuum. The yellow-brown solid was purified by column chromatography to give a yield of 68%; C_9_H_9_BrN_2_OS; ^1^H-NMR (CDCl_3_, 300 MHz): δ = 3.57 (t, *J* = 6.28 Hz, 2H, OCH_2_C*H*_2_Br), 4.22 (t, *J* = 6.28 Hz, 2H, OC*H*_2_CH_2_Br), 5.18 (s, br, 2H, Benzthia-2-N*H*_2_), 6.86 (d, *J* = 8.78 Hz, 1H, Benzthia-5-*H*), 7.09 (d, ^4^*J* = 2.55 Hz, 1H, Benzthia-7-*H*), 7.37 (d, *J* = 8.77 Hz, 1H, Benzthia-4-*H*); ESI-MS (m/z): 274.7 (M-H^+^, 100), 273.7 (M-H^+^, 12), 272.9 (M-H^+^, 94).

##### 2-amino-6-(3-bromopropoxy)benzothiazole (24b)

Intermediate **24b** was synthesized according to above described procedure for **24a**, however, starting from **23b** (4.2 mmol). Brown solid; yield: 45%; C_10_H_11_BrN_2_OS; ^1^H-NMR (CDCl_3_, 400 MHz): δ = 2.22–2.27 (m, 2H, OCH_2_C*H*_2_CH_2_Br) 3.55 (t, *J* = 6.42 Hz, 2H, OCH_2_ CH_2_C*H*_2_Br), 4.05 (t, *J* = 5.80 Hz, 2H, OC*H*_2_CH_2_CH_2_Br), 5.05 (s, br, 2H, Benzthia-2-N*H*_2_), 6.85 (d, *J* = 8.75 Hz, 1H, Benzthia-5-*H*), 7.07 (d, ^4^*J* = 2.53 Hz, 1H, Benzthia-7-*H*), 7.38 (d, *J* = 8.78 Hz, 1H, Benzthia-4-*H*); ESI-MS (m/z): 288.7 (M-H^+^, 100), 286.7 (M-H^+^, 96), 289.8 (M-H^+^, 12).

##### 2-amino-6-(4-bromobutoxy)benzothiazole (24c)

Intermediate **24c** was synthesized according to above described procedure for **24a**, however, starting from **23c** (4.2 mmol). Brown solid; yield: 47%; C_11_H_13_BrN_2_OS; ^1^H-NMR (d_6_ DMSO, 250 MHz): δ = 1.77 – 1.98 (m, 4H, OCH_2_C*H*_2_C*H*_2_CH_2_Br) 3.59 (t, *J* = 6.58 Hz, 2H, OCH_2_CH_2_CH_2_C*H*_2_Br), 3.96 (t, *J* = 6.13 Hz, 2H, OC*H*_2_CH_2_CH_2_CH_2_Br), 6.79 (d, *J* = 8.72 Hz, 1H, Benzthia-5-*H*), 7.21 (d, *J* = 8.67 Hz, 1H, Benzthia-4-*H*), 7.20 (s, br, 2H, Benzthia-2-N*H*_2_), 7.27 (d, ^4^*J* = 2.53 Hz, 1H, Benzthia-7-*H*); ESI-MS (m/z): 302.7 (M-H^+^, 100), 300.8 (M-H^+^, 95), 303.8 (M-H^+^, 13).

##### 2-amino-6-(2-(4-(2-methoxyphenyl)piperazin-1-yl) ethoxy)benzothiazole trihydrogenoxalate (3)

**24a** (0.6 mmol) was dissolved in acetone. 2-methoxyphenylpiperazine (0.6 mmol), anhydrous potassium carbonate (0.17 g, 1.2 mmol) and catalytic amounts of potassium iodide were added. The suspension was refluxed for 48 h, filtered and the yellow filtrate was evaporated to dry. The resulting yellowish raw product was purified by column chromatography to give a white product which was precipitated with anhydrous oxalic acid and dried. White solid (yield: 24%; mp: 162–163°C (decomp.); C_20_H_24_N_4_O_2_S × 3 C_2_H_2_O_4_; ^1^H-NMR (d_6_ DMSO, 300 MHz) δ = 3.10–3.30 (m, 4H, Pip-2-*H*, -6-*H*), 3.35–3.45 (m, 4H, Pip-3-*H*, 5-*H*), 3.53 (t, *J* = 4.81 Hz, 2H, OCH_2_C*H*_2_), 3.79 (s, 3 H, OC*H*_3_), 4.34 (t, *J* = 4.48 Hz, 2H, OC*H*_2_CH_2_), 6.91 (d, *J* = 8.12 Hz, 2H, 2-OCH_3_-Ar-3-*H*, -6-*H*), 6.98–7.04 (m, 2H, 2-OCH_3_-Ar-4-*H*, -5-*H*), 7.26 (d, *J* = 8.71 Hz, 1H, Benzthia-5-*H*), 7.34 (s, br, 2H, Benzthia-2-N*H*_2_), 7.38–7.41 (m, 2H, Benzthia-4-*H*, -7-*H*), 9.50 (s, br,2H, NH^+^); ^13^C-NMR (d_6_DMSO, 75 MHz) δ = 47.16 (Pip-3-*C*, -5-*C*), 51.96 (Pip-2-*C*, -6-*C*), 54.77 (OCH_2_*C*H_2_), 55.36 (O*C*H_3_), 63.27 (O*C*H_2_CH_2_), 106.95 (Benzthia-7-*C*), 111.98 (Benzthia-5-*C*), 113.74 (2-OCH_3_-Ar-*C*-4), 117.98 (2-OCH_3_-Ar-*C*-3), 118.24 (2-OCH_3_-Ar-*C*-6), 120.83 (2-OCH_3_-Ar-*C*-5), 123.34 (Benzthia-4-*C*), 131.76 (Benzthia-7a-*C*), 139.55 (Benzthia-3a-*C*), 147.27 (2-OCH_3_-Ar-*C*-1), 151.87 (2-OCH_3_-Ar-*C*-2), 152.53 (Benzthia-6-*C*), 162.28 ((*C*OOH)_2_), 165.14 (Benzthia-2-*C*), ESI-MS (m/z): 385.2 (M-H^+^, 100), 386.0 (M-H^+^, 22), 387.0 (M-H^+^, 7). CHN calc.: C 47.71, H 4.62, N 8.56; CHN found: C 47.45,H 4.82, N 8.30.

##### 2-amino-6-(3-(4-(2-methoxyphenyl)piperazin-1-yl) propoxy)benzothiazole (6)

**24b** (0.6 mmol) was dissolved in acetone. 2-methoxyphenylpiperazine (0.6 mmol), anhydrous potassium carbonate (0.17 g, 1.2 mmol) and catalytic amounts of potassium iodide were added. The suspension was refluxed for 48 h, filtered and the yellow filtrate was evaporated to dry. The resulting yellowish raw product was purified by column chromatography to give a white product which was precipitated with anhydrous oxalic acid and dried. White solid (yield: 82%; mp: 171°C); C_21_H_26_N_4_O_2_S; ^1^H-NMR (d_6_ DMSO, 300 MHz): δ = 1.83–1.92 (m, 2H, OCH_2_C*H*_2_CH_2_), 2.45–2.51 (m, 6H, Pip-2-*H*, -6-*H*, OCH_2_ CH_2_C*H*_2_), 2.96 (m, 4H, Pip-3-*H*, -5-*H*), 3.76 (s, 3 H, OC*H*_3_), 3.99 (t; *J* = 6.33 Hz, 2H, OC*H*_2_CH_2_CH_2_), 6.81 (d, *J* = 8.71 Hz, 1H, Benzthia-5-*H*), 6.85–6.94 (m, 4H, 2-OCH_3_-Ar-3-*H*, -4-*H*, -5-*H*, -6-*H*), 7.20 (s, br, 2H, Benzthia-2-N*H*_2_), 7.22 (d, *J* = 8.81 Hz, 1H, Benzthia-4-*H*), 7.29 (d, ^4^*J* = 2.57 Hz, 1H, Benzthia-*7*-H); ^13^C-NMR (d_6_ DMSO, 75 MHz): δ = 26.29 (OCH_2_*C*H_2_CH_2_), 50.04 (Pip-3-*C*, -5-*C*), 53.05 (Pip-2-*C*, -6-*C*), 54.51 (OCH_2_CH_2_*C*H_2_), 55.28 (O*C*H_3_), 66.44 (O*C*H_2_CH_2_CH_2_), 106.33 (Benzthia-7-*C*), 111.95 (Benzthia-5-*C*), 113.47 (2-OCH_3_-Ar-*C*-4), 117.85 (2-OCH_3_-Ar-*C*-3), 118.03 (2-OCH_3_-Ar-*C*-6), 120.82 (2-OCH_3_-Ar-*C*-5), 122.26 (Benzthia-4-*C*), 131.88 (Benzthia-7a-*C*), 141.27 (Benzthia-3a-*C*), 146.83 (2-OCH_3_-Ar-*C*-1), 151.96 (2-OCH_3_-Ar-*C*-2), 153.59 (Benzthia-6-*C*), 164.69 (Benzthia-2-*C*); ESI-MS (m/z): 399.0 (M-H^+^, 100), 400.0 (M-H^+^, 26), 401.0 (M-H^+^, 8). CHN calc.: C 63.29, H 6.58, N 14.06; CHN found: C 63.01, H 6.56, N 13.91.

##### 2-amino-6-(4-(4-(2-methoxyphenyl)piperazin-1-yl)butoxy) benzothiazole dihydrogenoxalate (9)

**24c** (0.6 mmol) was dissolved in acetone. 2-methoxyphenylpiperazine (0.6 mmol), anhydrous potassium carbonate (0.17 g, 1.2 mmol) and catalytic amounts of potassium iodide were added. The suspension was refluxed for 48 h, filtered and the yellow filtrate was evaporated to dry. The resulting yellowish raw product was purified by column chromatography to give a white product which was precipitated with anhydrous oxalic acid and dried. White solid [yield: 56%; mp 147–150°C (decomp.)]; C_22_H_28_N_4_O_2_S × 2 C_2_H_2_O_4_ × 0.25 H_2_O; ^1^H-NMR (d_6_ DMSO, 300 MHz): δ = 1.57–1.75 (m, 4H, OCH_2_C*H*_2_C*H*_2_CH_2_), 2.39 (t, *J* = 6.81 Hz, 2H, OCH_2_CH_2_CH_2_C*H*_2_), 2.49–2.51 (m, 4H, Pip-2-*H*, -6-*H*), 2.94 (m, 4H, Pip-3-*H*, -5-*H*), 3.76 (s, 3 H, OC*H*_3_), 3.96 (t, *J* = 6.32 Hz, 2H, OC*H*_2_CH_2_CH_2_CH_2_), 6.81 (d, *J* = 8.72 Hz, 1H, Benzthia-5-*H*), 6.85–6.94 (m, 4H, 2-OCH_3_-Ar-3-*H*, -4-*H*, -5-*H*, -6-*H*), 7.19 (s, br, 2H, Benzthia-2-N*H*_2_), 7.22 (d, *J* = 8.75 Hz, 1H, Benzthia-4-*H*), 7.28 (d, ^4^*J* = 2.56 Hz, 1H, Benzthia-*7*-H); ^13^C-NMR (d_6_ DMSO, 75 MHz): δ = 20.37 (OCH_2_CH_2_*C*H_2_CH_2_), 25.44 (OCH_2_*C*H_2_CH_2_CH_2_), 47.11 (Pip-3-*C*, -5-*C*), 51.27 (Pip-2-*C*, -6-*C*), 55.36 (OCH_2_CH_2_CH_2_*C*H_2_), 55.36 (O*C*H_3_), 67.44 (O*C*H_2_CH_2_CH_2_CH_2_), 106.46 (Benzthia-7-*C*), 111.96 (Benzthia-5-*C*), 113.52 (2-OCH_3_-Ar-4-*C*), 118.00 (2-OCH_3_-Ar-3-*C*), 118.24 (2-OCH_3_-Ar-6-*C*), 120.83 (2-OCH_3_-Ar-5-*C*), 123.38 (Benzthia-4-*C*), 131.82 (Benzthia-7a-*C*), 139.47 (Benzthia-3a-*C*), 146.84 (2-OCH_3_-Ar-1-*C*), 151.85 (2-OCH_3_-Ar-2-*C*), 153.41 (Benzthia-6-*C*), 162.79 ((*C*OOH)_2_), 164.77 (Benzthia-2-*C*); ESI-MS (m/z): 413.2 (M-H^+^, 100), 414.1 (M-H^+^, 27), 415.0 (M-H^+^, 8). CHN calc.: C 52.69, H 5.44, N 9.45; CHN found: C 52.32, H 5.77, N 9.29.

##### 2-amino-6-(2-(4-phenylpiperazin-1-yl)ethoxy)benzothiazole (12)

**24a** (0.6 mmol) was dissolved in acetone. *N*-phenylpiperazine (0.6 mmol), anhydrous potassium carbonate (0.17 g, 1.2 mmol) and catalytic amounts of potassium iodide were added. The suspension was refluxed for 48 h, filtered and the yellow filtrate was evaporated to dry. The resulting yellowish raw product was purified by column chromatography to give a yellow product. Yellow solid; yield: 61%; mp 159 °C; C_19_H_22_N_4_OS; ^1^H-NMR (d_6_ DMSO, 300 MHz): δ = 2.61–2.65 (m, 4H, Pip-2-*H*, -6-*H*), 2.74 (t, *J* = 5.73 Hz, 2 H, OCH_2_C*H*_2_), 3.11–3.14 (m, 4H, Pip-3-*H*, -5-*H*), 4.09 (t; *J* = 5.77 Hz, 2H, OC*H*_2_CH_2_), 6.76 (t, *J* = 7.25 Hz, 1H, Ar-4-*H*), 6.83 (d, *J* = 8.72 Hz, 1H, Benzthia-5-*H*), 6.92 (d, *J* = 8.81 Hz, 2H, Ar-2-*H*, -6-*H*), 7.18 (d, *J* = 7.32 Hz, 1H, Benzthia-4-*H*), 7.21 (s, br, 2H, Benzthia-2-N*H*_2_), 7.20–7.24 (m, 2H, Ar-3-*H*, -5-*H*), 7.32 (d, ^4^*J* = 2.57 Hz, 1H, Benzthia-*7*-*H*); ^13^C-NMR (d_6_ DMSO, 75 MHz) δ = 48.16 (Pip-3-*C*, -5-*C*), 53.04 (Pip-2-*C*, -6-*C*), 56.67 (OCH_2_*C*H_2_), 66.24 (O*C*H_2_CH_2_), 106.42 (Benzthia-7-*C*), 113.50 (Benzthia-5-*C*), 115.29 (Ar-*C*-2,-*C*-6), 118.02 (Ar-*C*-4), 118.70 (Benzthia-4-*C*), 128.85 (Ar-*C*-3, -*C*-5), 131.87 (Benzthia-7a-*C*), 146.91 (Benzthia-3a-*C*), 151.00 (Ar-*C*-1), 153.41 (Benzthia-6-*C*), 164.73 (Benzthia-2-*C*); ESI-MS (m/z): 355.0 (M-H^+^, 100), 356.0 (M-H^+^, 24), 357.1 (M-H^+^, 7). CHN calc.: C 64.38, H 6.26, N 15.81; CHN found: C 64.33, H 6.46, N 15.59.

##### 2-amino-6-(3-(4-phenylpiperazin-1-yl)propoxy) benzothiazole (15)

**24b** (0.6 mmol) was dissolved in acetone. *N*-phenylpiperazine (0.6 mmol), anhydrous potassium carbonate (0.17 g, 1.2 mmol) and catalytic amounts of potassium iodide were added. The suspension was refluxed for 48 h, filtered and the yellow filtrate was evaporated to dry. The resulting yellowish raw product was purified by column chromatography to give a white product. White solid; yield: 11%; mp 203°C; C_20_H_24_N_4_OS × 0.5 H_2_O; ^1^H-NMR (d_6_ DMSO, 300 MHz): δ = 1.90 (p, *J* = 6.54 Hz, 2H, OCH_2_C*H*_2_CH_2_), 2.44–2.53 (m, 6H, Pip-2-*H*, -6-*H*, OCH_2_ CH_2_C*H*_2_), 3.10–3.13 (m, 4H, Pip-3-*H*, -5-*H*), 3.99 (t; *J* = 6.33 Hz, 2H, OC*H*_2_CH_2_CH_2_), 6.73–6.82 (m, 2H, Benzthia-5-*H*, Ar-4-*H*), 6.92 (d, *J* = 7.96 Hz, 2H, Ar-2-*H*, -6-*H*), 7.17 – 7.23 (m, 3H, Ar-3-*H*, -5-*H*, Benzthia-4-*H*), 7.20 (s, br, 2H, Benzthia-2-N*H*_2_), 7.29 (d, ^4^*J* = 2.57 Hz, 1H, Benzthia-*7*-H); ^13^C-NMR (d_6_ DMSO, 75 MHz): δ = 26.30 (OCH_2_*C*H_2_CH_2_), 48.18 (Pip-3-*C*, -5-*C*), 52.77 (Pip-2-*C*, -6-*C*), 54.44 (OCH_2_CH_2_*C*H_2_), 66.46 (O*C*H_2_CH_2_CH_2_), 106.34 (Benzthia-7-*C*), 113.48 (Benzthia-5-*C*), 115.27 (Ar-2-*C*, -6-*C*), 118.02 (Ar-4-*C*), 118.86 (Benzthia-4-*C*), 128.85 (Ar-3-*C*, -5-*C*), 131.87 (Benzthia-7a-*C*), 146.86 (Benzthia-3a-*C*), 151.01 (Ar-1-*C*), 153.57 (Benzthia-6-*C*), 164.69 (Benzthia-2-*C*); ESI-MS (m/z): 369.0 (M-H^+^, 100), 370.1 (M-H^+^, 25), 371.0 (M-H^+^, 7). CHN calc.: C 63.63, H 6.68, N 14.84; CHN found: C 63.75, H 6.51, N 14.48.

##### 2-amino-6-(4-(4-phenylpiperazin-1-yl)butoxy)benzothiazole (18)

**24c** (0.6 mmol) was dissolved in acetone. *N*-phenylpiperazine (0.6 mmol), anhydrous potassium carbonate (0.17 g, 1.2 mmol) and catalytic amounts of potassium iodide were added. The suspension was refluxed for 48 h, filtered and the yellow filtrate was evaporated to dry. The resulting yellowish raw product was purified by column chromatography to give a white product. White solid; yield 26%; mp 164°C; C_21_H_26_N_4_OS × 0.25 H_2_O; ^1^H-NMR (d_6_ DMSO, 300 MHz): δ = 1.62–1.78 (m, 4H, OCH_2_C*H*_2_C*H*_2_CH_2_), 2.42 (t, *J* = 7.09 Hz, 2H, OCH_2_CH_2_CH_2_C*H*_2_), 2.53–2.56 (m, 4H, Pip-2-*H*, -6-*H*), 3.14–3.17 (m, 4H, Pip-3-*H*, -5-*H*), 4.02 (t, *J* = 6.36 Hz, 2H, OC*H*_2_CH_2_CH_2_CH_2_), 6.81 (t, *J* = 7.25 Hz, 1H, Ar-4-*H*), 6.86 (d, *J* = 8.75 Hz, 1H, Benzthia-5-*H*), 6.97 (d, *J* = 8.80 Hz, 2H, Ar-2-*H*, -6-*H*), 7.22–7.27 (m, 2H, Ar-3-*H*, -5-*H*), 7.25 (s, br, 2H, Benzthia-2-N*H*_2_), 7.27 (d, *J* = 8.80 Hz, 1H, Benzthia-4-*H*), 7.33 (d, ^4^*J* = 2.58 Hz, 1H, Benzthia-7-*H*); ^13^C-NMR (d_6_ DMSO, 75 MHz): δ = 22.73 (OCH_2_CH_2_*C*H_2_CH_2_), 26.66 (OCH_2_*C*H_2_CH_2_CH_2_), 48.19 (Pip-3-*C*, -5-*C*), 52.68 (Pip-2-*C*, -6-*C*), 57.33 (OCH_2_CH_2_CH_2_*C*H_2_), 67.91 (O*C*H_2_CH_2_CH_2_CH_2_), 106.32 (Benzthia-7-*C*), 113.45 (Benzthia-5-*C*), 115.25 (Ar-2-*C*, -6-*C*), 118.02 (Ar-4-*C*), 118.66 (Benzthia-4-*C*), 128.84 (Ar-3-*C*, -5-*C*), 131.86 (Benzthia-7a-*C*), 146.78 (Benzthia-3a-*C*), 151.03 (Ar-1-*C*), 153.58 (Benzthia-6-*C*), 164.65 (Benzthia-2-*C*); ESI-MS (m/z): 383.2 (M-H^+^, 100), 384.0 (M-H^+^, 26), 385.1 (M-H^+^, 8). CHN calc.: C 65.17, H 6.90, N 14.48; CHN found: C 64.98, H 6.78, N 14.26.

##### 6-(2-(4-(2-methoxyphenyl)piperazin-1-yl) ethoxy)benzothiazole hydrogenoxalate (4)

White solid; yield: 10%; mp 183°C; C_20_H_23_N_3_O_2_S × 1.5 C_2_H_2_O_4_ × 0.5 H_2_O; ^1^H-NMR (d_6_ DMSO, 400 MHz): δ = 3.20 (m, 4H, Pip-2-*H*, -6-*H*), 3.30 (m, 4H, Pip-3-*H*, -5-*H*), 3.45 (m, 2H, OCH_2_C*H*_2_), 3.80 (s, 3 H, OC*H*_3_), 4.43 (m, 2H, OC*H*_2_CH_2_), 6.88–7.03 (m, 4H, 2-OCH_3_-Ar-3-*H*, -4-*H*, -5-*H*, -6-*H*), 7.22 (d, *J* = 8.53 Hz, 1H, Benzthia-5-*H*), 7.81 (s, 1H, Benzthia-7-*H*), 8.01 (d, *J* = 8.80 Hz, 1H, Benzthia-4-*H*), 9.23 (s, 1H, Benzthia-2-*H*); ^13^C-NMR (d_6_DMSO, 100 MHz): δ = 47.37 (Pip-3-*C*, -5-*C*), 52.20 (Pip-2-*C*, -6-*C*), 55.02 (OCH_2_*C*H_2_), 55.38 (O*C*H_3_), 63.73 (O*C*H_2_CH_2_), 105.97 (Benzthia-7-*C*), 111.97 (Benzthia-5-*C*), 116.09 (2-OCH_3_-Ar-*C*-6), 118.20 (2-OCH_3_-Ar-*C*-3), 120.85 (2-OCH_3_-Ar-*C*-4), 123.20 (2-OCH_3_-Ar-*C*-5), 123.58 (Benzthia-4-*C*), 134.98 (Benzthia-7a-*C*), 139.89 (2-OCH_3_-Ar-*C*-1), 147.91 (Benzthia-3a-*C*), 151.90 (2-OCH_3_-Ar-*C*-2), 153.79 (Benzthia-2-*C*), 155.94 (Benzthia-6-*C*), 162.86 ((*C*OOH)_2_); ESI-MS: 370.0 (M-H^+^, 100), 371.0 (M-H^+^, 25), 372.0 (M-H^+^, 8). CHN calc.: C 53.79, H 5.30, N 8.18; CHN found: C 53.92, H 5.24, N 8.16.

##### 2-chloro-6-(2-(4-(2-methoxyphenyl)piperazin-1-yl) ethoxy)benzothiazole hydrogenoxalate (5)

In a three-neck round bottom flask anhydrous copper(II)chloride (0.65 g, 4.8 mmol) was suspended in 25 ml of dry acetonitrile. *t*-Butylnitrite (0.62 g, 6.0 mmol) was added and stirred at room temperature for 10 min to build a black suspension. **3** (4.0 mmol) was dissolved in a mixture of 25 ml dry acetonitrile and 10 ml dry methanol and added dropwise to the black suspension in the flask. The suspension was stirred 1.5 h at room temperature and 2.5 h at 65°C. After the suspension was evaporated with the addition of silica gel to afford a dry yellow-brown solid which was purified by column chromatography to provide a white product that was precipitated with anhydrous oxalic acid and dried. White solid; yield: 7%; mp 183–185°C (decomp.); C_20_H_22_ClN_3_O_2_S × 1 C_2_H_2_O_4_ × 0.5 H_2_O; ^1^H-NMR (d_6_ DMSO, 400 MHz): δ = 3.27 (m, 4H, Pip-2-*H*, -6-*H*), 3.37 (m, 4H, Pip-3-*H*, -5-*H*), 3.53 (m, 2H, OCH_2_C*H*_2_), 3.85 (s, 3H, OC*H*_3_), 4.47 (m, 2H, OC*H*_2_CH_2_), 6.94–7.09 (m, 4H, 2-OCH_3_-Ar-3-*H*, -4-*H*, -5-*H*, -6-*H*), 7.29 (d, *J* = 8.55 Hz, 1H, Benzthia-5-*H*), 7.83 (s, 1H, Benzthia-7-*H*), 7.96 (d, *J* = 8.80 Hz, 1H, Benzthia-4-*H*); ^13^C-NMR (d_6_ DMSO, 100 MHz): δ = 47.66 (Pip-3-*C*, -5-*C*), 52.21 (Pip-2-*C*, -6-*C*), 54.94 (OCH_2_*C*H_2_), 55.38 (O*C*H_3_), 63.97 (O*C*H_2_CH_2_), 106.23 (Benzthia-7-*C*), 111.99 (Benzthia-5-*C*), 116.49 (2-OCH_3_-Ar-*C*-6), 118.21 (2-OCH_3_-Ar-*C*-3), 120.85 (2-OCH_3_-Ar-*C*-4), 123.12 (2-OCH_3_-Ar-*C*-5), 123.21 (Benzthia-4-*C*), 137.05 (Benzthia-7a-*C*), 139.83 (2-OCH_3_-Ar-*C*-1), 145.09 (Benzthia-3a-*C*), 149.90 (2-OCH_3_-Ar-*C*-2), 151.90 (Benzthia-2-*C*), 156.13 (Benzthia-6-*C*), 162.86 ((*C*OOH)_2_); ESI-MS (m/z): 404.0 (M-H^+^, 100), 405.5 (M-H^+^, 36), 406.0 (M-H^+^, 40). CHN calc.: C 52.54, H 5.01, N 8.35; CHN found: C 52.49, H 4.94, N 8.41.

##### 6-(3-(4-(2-methoxyphenyl)piperazin-1-yl)propoxy) benzothiazole hydrogenoxalat (7)

White solid; yield: 35%; mp 168–170°C (decomp.); C_21_H_25_N_3_O_2_S × 1.5 C_2_H_2_O_4_; ^1^H-NMR (d_6_ DMSO, 300 MHz): δ = 2.17 – 2.23 (m, 2H, OCH_2_C*H*_2_CH_2_), 3.20–3.41 (m, 10H, Pip-2-*H*, -3-*H*, -5-*H*, -6-*H*, OCH_2_ CH_2_C*H*_2_), 3.79 (s, 3 H, OC*H*_3_), 4.16 (t; *J* = 5.85 Hz, 2H, OC*H*_2_CH_2_CH_2_), 6.90–7.03 (m, 4H, 2-OCH_3_-Ar-3-*H*, -4-*H*, -5-*H*, -6-*H*), 7.16 (d, *J* = 8.93 Hz, 1H, Benzthia-5-*H*), 7.74 (d, ^4^*J* = 2.47 Hz, 1H, Benzthia-7-*H*), 7.99 (d, *J* = 8.92 Hz, 1H, Benzthia-4-*H*), 9.19 (s, 1H, Benzthia-2-*H*); ^13^C-NMR (d_6_ DMSO, 75 MHz): δ = 23.47 (OCH_2_*C*H_2_CH_2_), 47.10 (Pip-3-*C*, -5-*C*), 51.41 (Pip-2-*C*, -6-*C*), 53.15 (OCH_2_CH_2_*C*H_2_), 55.37 (O*C*H_3_), 65.61 (O*C*H_2_CH_2_CH_2_), 105.70 (Benzthia-7-*C*), 112.00 (Benzthia-5-*C*), 115.98 (2-OCH_3_-Ar-*C*-6), 118.29 (2-OCH_3_-Ar-*C*-3), 120.84 (2-OCH_3_-Ar-*C*-4), 123.42 (2-OCH_3_-Ar-*C*-5), 123.46 (Benzthia-4-*C*), 134.98 (Benzthia-7a-*C*), 139.41 (2-OCH_3_-Ar-*C*-1), 147.71 (Benzthia-3a-*C*), 151.87 (2-OCH_3_-Ar-*C*-2), 153.52 (Benzthia-2-*C*), 156.38 (Benzthia-6-*C*), 162.06 ((*C*OOH)_2_); ESI-MS (m/z): 384.1 (M-H^+^, 100), 385.1 (M-H^+^, 24), 386.1 (M-H^+^, 9). CHN calc.: C 55.59, H 5.44, N 8.10; CHN found: C 55.44, H 5.52, N 8.11.

##### 2-chloro-6-(3-(4-(2-methoxyphenyl)piperazin-1-yl) propoxy)benzothiazole hydrogenoxalate (8)

In a three-neck round bottom flask anhydrous copper(II)chloride (0.65 g, 4.8 mmol) was suspended in 25 ml of dry acetonitrile. *t*-Butylnitrite (0.62 g, 6.0 mmol) was added and stirred at room temperature for 10 min to build a black suspension. **6** (4.0 mmol) was dissolved in a mixture of 25 ml dry acetonitrile and 10 ml dry methanol and added dropwise to the black suspension in the flask. The suspension was stirred 1.5 h at room temperature and 2.5 h at 65°C. Silica gel was added, and the suspension was evaporated with to provide a dry yellow-brown solid which was purified by column chromatography to provide a white product. Subsequently, the product was precipitated with anhydrous oxalic acid and dried. White solid; yield: 15%; mp 188°C; C_21_H_24_ClN_3_O_2_S × 1 C_2_H_2_O_4_ × 0.25 H_2_O; ^1^H-NMR (d_6_ DMSO, 300 MHz): δ = 2.17–2.24 (m, 2H, OCH_2_C*H*_2_CH_2_), 3.23–3.44 (m, 10H, Pip-2-*H*, -3-*H*, -5-*H*, -6-*H*, OCH_2_ CH_2_C*H*_2_), 3.79 (s, 3H, OC*H*_3_), 4.14 (t; *J* = 5.64 Hz, 2H, OC*H*_2_CH_2_CH_2_), 6.90–7.02 (m, 4H, 2-OCH_3_-Ar-3-*H*, -4-*H*, -5-*H*, -6-*H*), 7.16 (d, *J* = 8.95 Hz, 1H, Benzthia-5-*H*), 7.70 (d, ^4^*J* = 2.55 Hz, 1H, Benzthia-7-*H*), 7.88 (d, *J* = 8.95 Hz, 1H, Benzthia-4-*H*); ^13^C-NMR (d_6_ DMSO, 75 MHz): δ = 23.50 (OCH_2_*C*H_2_CH_2_), 47.19 (Pip-3-*C*, -5-*C*), 51.41 (Pip-2-*C*, -6-*C*), 53.12 (OCH_2_CH_2_*C*H_2_), 55.36 (O*C*H_3_), 65.67 (O*C*H_2_CH_2_CH_2_), 105.99 (Benzthia-7-*C*), 112.00 (Benzthia-5-*C*), 116.36 (2-OCH_3_-Ar-*C*-6), 118.28 (2-OCH_3_-Ar-*C*-3), 120.84 (2-OCH_3_-Ar-*C*-4), 123.05 (2-OCH_3_-Ar-*C*-5), 123.40 (Benzthia-4-*C*), 137.06 (Benzthia-7a-*C*), 139.46 (2-OCH_3_-Ar-*C*-1), 144.86 (Benzthia-3a-*C*), 149.61 (2-OCH_3_-Ar-*C*-2), 151.87 (Benzthia-2-*C*), 156.61 (Benzthia-6-*C*), 162.21 ((*C*OOH)_2_); ESI-MS (m/z): 418.1 (M-H^+^, 100), 419.4 (M-H^+^, 36), 420.1 (M-H^+^, 40). CHN calc.: C 53.90, H 5.21, N 8.20; CHN found: C 53.97, H 5.35, N 8.22.

##### 6-(4-(4-(2-methoxyphenyl)piperazin-1-yl)butoxy) benzothiazole hydrogenoxalat (10)

White solid; yield: 36%; mp 159–165°C (decomp.); C_22_H_27_N_3_O_2_S × 1.5 C_2_H_2_O_4;_^1^H-NMR (d_6_ DMSO, 300 MHz): δ = 1.83 (m, 4H, OCH_2_C*H*_2_C*H*_2_CH_2_), 3.13–3.37 (m, 10H, OCH_2_CH_2_CH_2_C*H*_2_, Pip-2-*H*, -3-*H*, -5-*H*, -6-*H*), 3.79 (s, 3H, OC*H*_3_), 4.10 (t, *J* = 5.13 Hz, 2H, OC*H*_2_CH_2_CH_2_CH_2_), 6.87–7.02 (m, 4H, 2-OCH_3_-Ar-3-*H*, -4-*H*, -5-*H*, -6-*H*), 7.15 (d, *J* = 8.92 Hz, 1H, Benzthia-5-*H*), 7.73 (d, ^4^*J* = 2.37 Hz, 1H, Benzthia-*7*-H), 7.97 (d, *J* = 8.92 Hz, 1H, Benzthia-4-*H*), 9.19 (s, 1H, Benzthia-2-*H*); ^13^C-NMR (d_6_ DMSO, 75 MHz): δ = 19.98 (OCH_2_CH_2_*C*H_2_CH_2_), 26.13 (OCH_2_*C*H_2_CH_2_CH_2_), 46.88 (Pip-3-*C*, -5-*C*), 51.49 (Pip-2-*C*, -6-*C*), 55.35 (OCH_2_CH_2_CH_2_*C*H_2_), 55.35 (O*C*H_3_), 67.37 (O*C*H_2_CH_2_CH_2_CH_2_), 105.53 (Benzthia-7-*C*), 111.95 (Benzthia-5-*C*), 115.99 (2-OCH_3_-Ar-3-*C*), 115.99 (2-OCH_3_-Ar-6-*C*), 118.23 (2-OCH_3_-Ar-4-*C*), 120.82 (2-OCH_3_-Ar-5-*C*), 123.33 (Benzthia-4-*C*), 134.99 (Benzthia-7a-*C*), 139.47 (Benzthia-3a-*C*), 147.56 (2-OCH_3_-Ar-1-*C*), 151.86 (2-OCH_3_-Ar-2-*C*), 153.34 (Benzthia-2-*C*), 156.66 (Benzthia-6-*C*), 163.05 ((*C*OOH)_2_); ESI-MS (m/z): 398.1 (M-H^+^, 100), 399.1 (M-H^+^, 26), 400.1 (M-H^+^, 8). CHN calc.: C 56.38, H 5.68, N 7.89; CHN found: C 56.12, H 5.84, N 7.88.

##### 6-(4-bromobutoxy)benzothiazole (24d)

Yellow oil; yield: 54%; C_11_H_12_BrNOS; ^1^H-NMR (CDCl_3_, 400 MHz): δ = 1.98–2.09 (m, 4H, OCH_2_C*H*_2_C*H*_2_CH_2_Br) 3.07 (t, *J* = 7.02 Hz, 2H, OCH_2_CH_2_CH_2_C*H*_2_Br), 4.09 (t, *J* = 5.78 Hz, 2H, OC*H*_2_CH_2_CH_2_CH_2_Br), 7.11 (d, *J* = 8.95 Hz, 1H, Benzthia-5-*H*), 7.38 (d, ^4^*J* = 2.44 Hz, 1H, Benzthia-7-*H*), 8.01 (d, *J* = 8.95 Hz, 1H, Benzthia-4-*H*), 8.83 (s, 1H, Benzthia-2-*H*); ESI-MS (m/z): 264.9 (M-SCN H^+^, 100), 265.9 (M-SCN H^+^, 15), 267.0 (M-SCN H^+^, 10).

##### 6-(4-(4-phenylpiperazin-1-yl)butoxy)benzothiazole dihydrogenoxalate (19)

**24d** (0.6 mmol) was dissolved in acetone. *N*-phenylpiperazine (0.09 g, 0.6 mmol), anhydrous potassium carbonate (0.17 g, 1.2 mmol) and catalytic amounts of potassium iodide were added. The suspension was refluxed for 48 h, filtered and the yellow filtrate was evaporated to dry. The resulting yellowish raw product was purified by column chromatography. The resulting colorless oil was precipitated with anhydrous oxalic acid and dried to give a white solid (yield: 17%); mp 168°C (decomp.); C_21_H_25_N_3_OS × 2 C_2_H_2_O_4_ × 0.25 H_2_O; ^1^H-NMR (d_6_ DMSO, 300 MHz) δ = 1.79–1.86 (m, 4H, OCH_2_C*H*_2_C*H*_2_CH_2_), 3.12–3.39 (m, 10H, OCH_2_CH_2_CH_2_C*H*_2_, Pip-2-*H*, -3-*H*, -5-*H*,-6-*H*), 4.10 (t, *J* = 5.61 Hz, 2H, OC*H*_2_CH_2_CH_2_CH_2_), 6.85 (t, *J* = 7.26 Hz, 1H, Ar-4-*H*), 6.99 (d, *J* = 7.89 Hz, 2H, Ar-2-*H*, -6-*H*), 7.15 (d, *J* = 8.93 Hz, 1H, Benzthia-5-*H*), 7.23–7.28 (m, 2H, Ar-3-*H*, -5-*H*), 7.72 (d, ^4^*J* = 2.48 Hz, 1H, Benzthia-7-*H*), 7.97 (d, *J* = 8.93 Hz, 1H, Benzthia-4-*H*), 9.19 (s, 1H, Benzthia-2-*H*); ^13^C-NMR (d_6_ DMSO, 75 MHz): δ = 20.41 (OCH_2_CH_2_*C*H_2_CH_2_), 25.84 (OCH_2_*C*H_2_CH_2_CH_2_), 45.70 (Pip-3-*C*, -5-*C*), 50.83 (Pip-2-*C*, -6-*C*), 55.31 (OCH_2_CH_2_CH_2_*C*H_2_), 67.45 (O*C*H_2_CH_2_CH_2_CH_2_), 105.50 (Benzthia-7-*C*), 115.85 (Ar-2-*C*, -6-*C*), 115.98 (Benzthia-5-*C*), 119.84 (Ar-4-*C*), 123.41 (Benzthia-4-*C*), 129.06 (Ar-3-*C*, -5-*C*), 134.99 (Benzthia-7a-*C*), 147.53 (Benzthia-3a-*C*), 149.67 (Ar-1-*C*), 153.36 (Benzthia-2-*C*), 156.64 (Benzthia-6-*C*), 162.82 ((*C*OOH)_2_); ESI-MS (m/z): 368.1 (M-H^+^, 100), 369.2 (M-H^+^, 25), 370.1 (M-H^+^, 8). CHN calc.: C 54.39, H 5.39, N 7.61; CHN found: C 54.37, H 5.22, N 7.74.

##### 1-(2-hydroxyethoxy)-4-nitrobenzene (25a)

Yellow solid; yield: 72%; C_8_H_9_NO_4;_^1^H-NMR (CDCl_3_, 300 MHz): δ = 3.96 (td, *J* = 4.60 Hz, 2H, OCH_2_C*H*_2_OH), 4.32 (td, *J* = 5.11 Hz, 2H, OC*H*_2_CH_2_OH), 6.93 (d, *J* = 9.28 Hz, 2H, Ar-2-*H*, -6-*H*), 8.16 (d, *J* = 9.27 Hz, 2H, Ar-3-*H*, -5-*H*); ESI-MS (m/z): 137.6 (4-nitrophenolate^−^, 100), 138.6 (4-nitrophenolate^−^, 8), 183.5 (M^−^, 4).

##### 1-(3-hydroxypropoxy)-4-nitrobenzene (25b)

Yellow oil; yield: 100%; C_9_H_11_${\text{NO}}_{4;}^{1}$H-NMR (CDCl_3_, 400 MHz): δ = 1.87 (p, *J* = 5.99 Hz, 2H, OCH_2_C*H*_2_CH_2_OH) 3.66 (t, *J* = 5.94 Hz, 2H, OCH_2_ CH_2_C*H*_2_OH), 4.01 (t, *J* = 6.10 Hz, 2H, OC*H*_2_CH_2_CH_2_OH), 6.75 (d, *J* = 9.22 Hz, 2H, Ar-2-*H*, -6-*H*), 7.97 (d, *J* = 9.21 Hz, 2H, Ar-3-*H*, -5-*H*); ESI-MS (m/z): 197.7 (M-H^+^, 100), 198.7 (M-H^+^, 21).

##### 1-(4-hydroxybutoxy)-4-nitrobenzene (25c)

**22c** (2.74 g, 1.0 mmol) was dissolved in 200 ml of a mixture acetone: water (7:3 v/v). After addition of the base *N,N*-diisopropylamine (1.1 g, 1.1 mmol) the solution was heated in a Parr apparatus to 130°C for 9 h under continuous steering. After cooling and evaporation of acetone, the brown solution was acidified with hydrochloric acid solution (2 N) and extracted with ethyl acetate. The combined organic layers were dried over sodium sulfate. After evaporation of the solvent the crude product was purified by column chromatography to give a white solid (0.89 g, yield: 45%); ^1^H-NMR (d_6_ DMSO, 300 MHz) δ = 1.51–1.60 (m, 2H, OCH_2_CH_2_C*H*_2_CH_2_OH), 1.73–1.80 (m, 2H, OCH_2_C*H*_2_CH_2_CH_2_OH), 3.42–3.48 (m, 2H, OCH_2_CH_2_CH_2_C*H*_2_OH), 4.13 (t, *J* = 6.55 Hz, 2H, OC*H*_2_CH_2_CH_2_CH_2_OH), 4.44 (t, *J* = 5.14 Hz, 1H, OCH_2_CH_2_CH_2_CH_2_O*H*), 7.12 (d, *J* = 9.32 Hz, 2H, Ar-2-*H*, -6-*H*), 8.19 (d, *J* = 9.32 Hz, 2H, Ar-3-*H*, -5-*H*); ESI-MS (m/z): 211.7 (M-H^+^, 100), 212.9 (M-H^+^, 23), 213.8 (M-H^+^, 13).

##### 4-(2-hydroxyethoxy)aniline (26a)

**25a** (4.2 mmol) was dissolved with 50 ml ethyl acetate. 100 mg Pd/C (10% m/m) and a few drops glacial acetic acid were added. The suspension was stirred at room temperature in a Parr apparatus with a pressure of 5 bar hydrogen for 1 h. After filtration of the catalyst the yellow-brown solution was evaporated to dryness to give brownish oil. Brown oil; yield: 100%; C_8_H_11_${\text{NO}}_{2;}^{1}$H-NMR (d_6_ DMSO, 300 MHz): δ = 3.63 (t, *J* = 5.19 Hz, 2H, OCH_2_C*H*_2_OH), 3.82 (t, *J* = 5.13 Hz, 2H, OC*H*_2_CH_2_OH), 6.49 (d, *J* = 8.97 Hz, 2H, Ar-2-*H*, -6-*H*), 6.64 (d, *J* = 8.92 Hz, 2H, Ar-3-*H*, -5-*H*); ESI-MS (m/z): 153.8 (M-H^+^, 100), 154.7 (M-H^+^, 12), 175.7 (M-Na^+^, 87).

##### 4-(3-hydroxypropoxy)-aniline (26b)

**25b** (4.2 mmol) was dissolved with 50 ml ethyl acetate. 100 mg Pd/C (10% m/m) and a few drops glacial acetic acid were added. The suspension was stirred at room temperature in a Parr apparatus with a pressure of 5 bar hydrogen for 1 h. After filtration of the catalyst the yellow-brown solution was evaporated to dryness to give brownish oil. Brown oil; yield: 100%; C_9_H_13_${\text{NO}}_{2;}^{1}$H-NMR (d_6_ DMSO, 300 MHz): δ = 1.79 (p, *J* = 6.34 Hz, 2H, OCH_2_C*H*_2_CH_2_OH) 3.53 (t, *J* = 6.30 Hz, 2H, OCH_2_ CH_2_C*H*_2_OH), 3.88 (t, *J* = 6.40 Hz, 2H, OC*H*_2_CH_2_CH_2_OH), 6.57 (d, *J* = 8.91 Hz, 2H, Ar-2-*H*, -6-*H*), 6.67 (d, *J* = 8.92 Hz, 2H, Ar-3-*H*, -5-*H*); ESI-MS (m/z): 167.7 (M-H^+^, 100), 168.7 (M-H^+^, 11).

##### 4-(4-hydroxybutoxy)aniline (26c)

**25c** (0.89 g, 4.2 mmol) was dissolved with 50 ml ethyl acetate. 100 mg Pd/C (10% m/m) and a few drops glacial acetic acid were added. The suspension was stirred at room temperature in a Parr apparatus with a pressure of 5 bar hydrogen for 1 h. After filtration of the catalyst the yellow-brown solution was evaporated to dryness to give a brownish oil (0.76 g, yield: 100%). Brown oil; C_10_H_15_${\text{NO}}_{2;}^{1}$H-NMR (d_6_ DMSO, 400 MHz) δ = 1.55–1.63 (m, 2H, OCH_2_CH_2_C*H*_2_CH_2_OH), 1.69–1.76 (m, 2H, OCH_2_C*H*_2_CH_2_CH_2_OH), 3.49 (t, *J* = 6.48 Hz, 2H, OCH_2_CH_2_CH_2_C*H*_2_OH), 3.87 (t, *J* = 6.50 Hz, 2H, OC*H*_2_CH_2_CH_2_CH_2_OH), 6.55 (d, *J* = 8.78 Hz, 2H, Ar-2-*H*, -6-*H*), 6.69 (d, *J* = 8.80 Hz, 2H, Ar-3-*H*, -5-*H*); ESI-MS (m/z): 181.8 (M-H^+^, 100), 182.7 (M-H^+^, 12).

##### 2-amino-6-(2-hydroxyethoxy)benzothiazole (27a)

**26a** (4.4 mmol) and potassium thiocyanate (1.71 g, 17.6 mmol) were solved in 25 ml glacial acetic acid. The resultant brown solution was cooled on an ice bath. Dropwise bromine (0.71 g, 4.4 mmol) dissolved in 5 ml glacial acetic acid was added, while the temperature was held constant at 4°C. Afterwards the orange suspension was stirred at room temperature overnight. The formed orange solid was filtered of and the yellow filtrate was concentrated in vacuum. The yellow-orange solid was purified by column chromatography to give a white solid (yield: 50%); C_9_H_10_N_2_O_2_S; ^1^H-NMR (d_6_ DMSO, 300 MHz): δ = 3.77 (t, *J* = 4.98 Hz, 2H, OCH_2_C*H*_2_OH), 4.02 (t, *J* = 5.03 Hz, 2H, OC*H*_2_CH_2_OH), 6.89 (d, *J* = 8.73 Hz, 1H, Benzthia-5-*H*), 7.29 (d, *J* = 8.71 Hz, 1H, Benzthia-4-*H*), 7.35 (s, br, 2H, Benzthia-2-N*H*_2_), 7.36 (s, 1H, Benzthia-7-*H*).

##### 2-amino-6-(3-hydroxypropoxy)benzothiazole (27b)

**26b** (4.4 mmol) and potassium thiocyanate (1.71 g, 17.6 mmol) were solved in 25 ml glacial acetic acid. The resultant brown solution was cooled on an ice bath. Dropwise bromine (0.71 g, 4.4 mmol) dissolved in 5 ml glacial acetic acid was added, while the temperature was held constant at 4°C. Afterwards the orange suspension was stirred at room temperature overnight. The resulted orange solid was filtered of and the yellow filtrate was concentrated in vacuum. The yellow-orange solid was purified by column chromatography to give a brownish oil (yield: 25%); C_10_H_12_N_2_O_2_S; ^1^H-NMR (d_6_ DMSO, 300 MHz): δ = 1.80–1.87 (m, 2H, OCH_2_C*H*_2_CH_2_OH) 3.54 (t, *J* = 6.14 Hz, 2H, OCH_2_ CH_2_C*H*_2_OH), 3.99 (t, *J* = 6.26 Hz, 2H, OC*H*_2_CH_2_CH_2_OH), 6.83 (d, *J* = 8.66 Hz, 1H, Benzthia-5-*H*), Benzthia-7-*H*), 7.23 (d, *J* = 8.70 Hz, 1H, Benzthia-4-*H*), 7.30 (d, ^4^*J* = 2.05 Hz, 1H, 7.54 (s, br, 2H, Benzthia-2-N*H*_2_); ESI-MS (m/z): 224.7 (M-H^+^, 100), 225.8 (M-H^+^, 12), 226.8 (M-H^+^, 6).

##### 2-amino-6-(4-hydroxybutoxy)benzothiazole (27c)

**26c** (0.76 g, 4.4 mmol) and potassium thiocyanate (1.71 g, 17.6 mmol) were solved in 25 ml glacial acetic acid. The resultant brown solution was cooled on an ice bath. Dropwise bromine (0.71 g, 4.4 mmol) dissolved in 5 ml glacial acetic acid was added, while the temperature was held constant at 4°C. Afterwards the orange suspension was stirred at room temperature overnight. The resulted orange solid was filtered of and the yellow filtrate was concentrated in vacuum. The yellow-orange solid was purified by column chromatography to give a white solid (0.96 g, yield: 91%); C_11_H_14_N_2_O_2_S; ^1^H-NMR (d_6_ DMSO, 400 MHz): δ = 1.52–1.59 (m, 2H, OCH_2_CH_2_C*H*_2_CH_2_OH), 1.69–1.76 (m, 2H, OCH_2_C*H*_2_CH_2_CH_2_OH), 3.43–3.45 (m, 2H, OCH_2_CH_2_CH_2_C*H*_2_OH), 3.94 (t, *J* = 6.43 Hz, 2H, OC*H*_2_CH_2_CH_2_CH_2_OH), 6.80 (d, *J* = 8.64 Hz, 1H, Benzthia-5-*H*), 7.19 (s, br, 2H, Benzthia-2-N*H*_2_), 7.21 (d, *J* = 8.95 Hz, 1H, Benzthia-4-*H*), 7.27 (d, ^4^*J* = 2.07 Hz, 1H, Benzthia-7-*H*); ESI-MS (m/z): 238.9 (M-H^+^, 100), 239.9 (M-H^+^, 13), 240.9 (M-H^+^, 6).

##### 6-(2-hydroxyethoxy)benzothiazole (28)

White solid; yield: 31%; C_9_H_9_NO_2_S; ^1^H-NMR (CDCl_3_, 300 MHz): δ = 3.91–3.96 (m, 2H, OCH_2_C*H*_2_OH), 4.08–4.11 (m, 2H, OC*H*_2_CH_2_OH), 7.07 (d, *J* = 8.96 Hz, 1H, Benzthia-5-*H*), 7.34 (d, ^4^*J* = 2.50 Hz,1H, Benzthia-7-*H*), 7.95 (d, *J* = 8.97 Hz, 1H, Benzthia-4-*H*), 8.77 (s, 1H, Benzthia-2-*H*); ESI-MS (m/z): 195.8 (M-H^+^, 100), 196.8 (M-H^+^, 11), 197.7 (M-H^+^, 5).

##### 6-(3-hydroxypropoxy)benzothiazole (29)

Brown oil; yield: 40%; C_10_H_11_NO_2_S; ^1^H-NMR (CDCl_3_, 400 MHz): δ = 2.02 (p, *J* = 5.96 Hz, 2H, OCH_2_C*H*_2_CH_2_OH) 3.83 (t, *J* = 5.91 Hz, 2H, OCH_2_ CH_2_C*H*_2_OH), 4.13 (t, *J* = 6.00 Hz, 2H, OC*H*_2_CH_2_CH_2_OH), 7.04 (d, *J* = 8.95 Hz, 1H, Benzthia-5-*H*), 7.32 (d, ^4^*J* = 2.43 Hz, 1H, Benzthia-7-*H*), 7.92 (d, *J* = 8.95 Hz, 1H, Benzthia-4-*H*), 8.75 (s, 1H, Benzthia-2-*H*); ^13^C-NMR (CDCl_3_): δ = 32.03 (OCH_2_*C*H_2_CH_2_), 60.10 (OCH_2_CH_2_*C*H_2_), 66.16 (O*C*H_2_CH_2_CH_2_), 104.90 (Benzthia-7-*C*), 116.18 (Benzthia-5-*C*), 124.02 (Benzthia-4-*C*), 135.11 (Benzthia-7a-*C*), 147.93 (Benzthia-3a-*C*), 151.55 (Benzthia-2-*C*), 157.27 (Benzthia-6-*C*); ESI-MS (m/z): 209.7 (M-H^+^, 100), 210.7 (M-H^+^, 12), 211.7 (M-H^+^, 5).

##### 2-chloro-6-(2-hydroxyethoxy)benzothiazole (30a)

In a three-neck round bottom flask anhydrous copper(II)chloride (0.65 g, 4.8 mmol) was suspended in 25 ml of dry acetonitrile. *t*-Butylnitrite (0.62 g, 6.0 mmol) was added and stirred at room temperature for 10 min to build a black suspension. **27a** (4.0 mmol) was dissolved in a mixture of 25 ml dry acetonitrile and 10 ml dry methanol and added dropwise to the black suspension in the flask. The suspension was stirred 1.5 h at room temperature and 2.5 h at 65°C. After the suspension was evaporated with the addition of silicagel to a dry yellow-brown solid, it was purified by column chromatography to yellowish oil (yield: 45%); C_9_H_8_ClNO_2_S; ^1^H-NMR (d_6_ DMSO, 250 MHz): δ = 3.73 (t, *J* = 4.76 Hz, 2H, OCH_2_C*H*_2_OH), 4.03 (t, *J* = 4.92 Hz, 2H, OC*H*_2_CH_2_OH), 7.14 (d, *J* = 8.96 Hz, 1H, Benzthia-5-*H*), 7.66 (d, ^4^*J* = 2.57 Hz,1H, Benzthia-7-*H*), 7.83 (d, *J* = 8.95 Hz, 1H, Benzthia-4-*H*); ESI-MS (m/z) 183.6 (2-chlorobenthiazole-6-O^−^, 100), 184.5 (2-chlorobenthiazole-6-O^−^, 9), 185.6 (2-chlorobenthiazole-6-O^−^, 37).

##### 2-chloro-6-(3-hydroxypropoxy)benzothiazole (30b)

In a three-neck round bottom flask anhydrous copper(II)chloride (0.65 g, 4.8 mmol) was suspended in 25 ml of dry acetonitrile. *t*-Butylnitrite (0.62 g, 6.0 mmol) was added and stirred at room temperature for 10 min to build a black suspension. **27b** (4.0 mmol) was dissolved in a mixture of 25 ml dry acetonitrile and 10 ml dry methanol and added dropwise to the black suspension in the flask. The suspension was stirred 1.5 h at room temperature and 2.5 h at 65°C. After the suspension was evaporated with the addition of silicagel to a dry yellow-brown solid, it was purified by column chromatography to yellowish oil (yield: 58%); C_10_H_10_ClNO_2_S; ^1^H-NMR (CDCl_3_, 400 MHz): δ = 2.02 (p, *J* = 5.93 Hz, 2H, OCH_2_C*H*_2_CH_2_OH) 3.82 (t, *J* = 5.89 Hz, 2H, OCH_2_ CH_2_C*H*_2_OH), 4.10 (t, *J* = 6.01 Hz, 2H, OC*H*_2_CH_2_CH_2_OH), 7.00 (d, *J* = 8.96 Hz, 1H, Benzthia-5-*H*), 7.17 (d, ^4^*J* = 2.46 Hz, 1H, Benzthia-7-*H*), 7.74 (d, *J* = 8.95 Hz, 1H, Benzthia-4-*H*); ESI-MS (m/z): 243.7 (M-H^+^, 100), 245.7 (M-H^+^, 46), 246.7 (M-H^+^, 5).

##### 2-chloro-6-(4-hydroxybutoxy)benzothiazole (30c)

In a three-neck round bottom flask anhydrous copper(II)chloride (0.65 g, 4.8 mmol) was suspended in 25 ml of dry acetonitrile. *t*-Butylnitrite (0.62 g, 6.0 mmol) was added and stirred at room temperature for 10 min to build a black suspension. **27c** (0.96 g, 4.0 mmol) was dissolved in a mixture of 25 ml dry acetonitrile and 10 ml dry methanol and added dropwise to the black suspension in the flask. The suspension was stirred 1.5 h at room temperature and 2.5 h at 65°C. After the suspension was evaporated with the addition of silicagel to a dry yellow-brown solid, it was purified by column chromatography to yellowish oil (0.5 g, yield: 48%); C_11_H_12_ClNO_2_S; ^1^H-NMR (d_6_ DMSO, 300 MHz): δ = 1.52–1.61 (m, 2H, OCH_2_CH_2_C*H*_2_CH_2_OH), 1.73–1.80 (m, 2H, OCH_2_C*H*_2_CH_2_CH_2_OH), 3.42–3.48 (m, 2H, OCH_2_CH_2_CH_2_C*H*_2_OH), 4.04 (t, *J* = 6.40 Hz, 2H, OC*H*_2_CH_2_CH_2_CH_2_OH), 4.45 (t, *J* = 4.74 Hz, 1H, OCH_2_CH_2_CH_2_CH_2_O*H*), 7.12 (d, *J* = 8.94 Hz, 1H, Benzthia-5-*H*), 7.66 (d, ^4^*J* = 2.58 Hz, 1H, Benzthia-7-*H*), 7.83 (d, *J* = 8.95 Hz, 1H, Benzthia-4-*H*); ESI-MS (m/z): 257.8 (M-H^+^, 100), 258.9 (M-H^+^, 13), 259.9 (M-H^+^, 39).

##### 2-(benzothiazol-6-yloxy)ethyl methanesulfonate (31a)

Yellow oil; yield: 77%; C_10_H_11_NO_4_${\text{S}}_{2;}^{1}$H-NMR (CDCl_3_, 300 MHz) δ = 3.04 (s, 3H, O-SO_2_-C*H*_3_), 4.24–4.27 (m, 2H, OCH_2_C*H*_2_O), 4.53–4.56 (m, 2H, OC*H*_2_CH_2_O), 7.07 (d, *J* = 8.96 Hz, 1H, Benzthia-5-*H*), 7.34 (d, ^4^*J* = 2.51 Hz,1H, Benzthia-7-*H*), 7.97 (d, *J* = 8.95 Hz, 1H, Benzthia-4-*H*), 8.80 (s, 1H, Benzthia-2-*H*); ESI-MS (m/z): 274.0 (M-H^+^, 100), 275.0 (M-H^+^, 13), 276.0 (M-H^+^, 11).

##### 2-(2-chlorobenzothiazol-6-yloxy)ethyl methanesulfonate (31b)

**30a** (0.5 g, 1.95 mmol) solved in 20 ml dichlormethane were added to triethylamine (0.3 g, 2.9 mmol). The solution was cooled under argon atmosphere to −10°C. Methanesulfonic chloride (0.27 g, 2.3 mmol) dissolved in 10 ml dichlormethane was added dropwise. The solution was stirred 15 min by −10°C and 45 min by room temperature, poured into ice water and twice extracted with a sodium carbonate solution. The organic layer was dried over sodium sulfate and evaporated to give a yellow oil (yield 100%); C_10_H_10_ClNO_4_${\text{S}}_{2;}^{1}$H-NMR (CDCl_3_, 250 MHz): δ = 3.03 (s, 3H, O-SO_2_-C*H*_3_), 4.23 (m, 2H, OCH_2_C*H*_2_O), 4.52–4.55 (m, 2H, OC*H*_2_CH_2_O), 7.02 (d, *J* = 8.95 Hz, 1H, Benzthia-5-*H*), 7.19 (s,1H, Benzthia-7-*H*), 7.78 (d, *J* = 8.96 Hz, 1H, Benzthia-4-*H*); ESI-MS (m/z): 307.8 (M-H^+^, 100), 308.9 (M-H^+^, 13), 309.8 (M-H^+^, 43).

##### 3-(benzothiazol-6-yloxy)propyl methanesulfonate (31c)

Brown oil; yield: 75%; C_11_H_13_NO_4_${\text{S}}_{2;}^{1}$H-NMR (CDCl_3_, 400 MHz): δ = 2.22 (p, *J* = 5.98 Hz, 2H, OCH_2_C*H*_2_CH_2_O), 2.94 (s, 3H, O-SO_2_-C*H*_3_), 4.11 (t, *J* = 5.87 Hz, 2H, OCH_2_ CH_2_C*H*_2_O), 4.42 (t, *J* = 6.09 Hz, 2H, OC*H*_2_CH_2_CH_2_O), 7.05 (d, *J* = 8.95 Hz, 1H, Benzthia-5-*H*), 7.34 (d, ^4^*J* = 2.43 Hz, 1H, Benzthia-7-*H*), 7.95 (d, *J* = 8.95 Hz, 1H, Benzthia-4-*H*), 8.77 (s, 1H, Benzthia-2-*H*); ESI-MS (m/z): 287.7 (M-H^+^, 100), 288.9 (M-H^+^, 13), 289.9 (M-H^+^, 10).

##### 3-(2-chlorobenzothiazol-6-yloxy)propyl methanesulfonate (31d)

**30b** (0.5 g, 1.95 mmol) solved in 20 ml dichlormethane were added to triethylamine (0.3 g, 2.9 mmol). The solution was cooled under argon atmosphere to −10°C. Methanesulfonic chloride (0.27 g, 2.3 mmol) dissolved in 10 ml dichlormethane was added dropwise. The solution was stirred 15 min by −10°C and 45 min by room temperature, poured into ice water and twice extracted with a sodium carbonate solution. The organic layer was dried over sodium sulfte and evaporated to give a yellow oil (yield 88%); C_11_H_12_ClNO_4_S_2_; ^1^H-NMR (CDCl_3_, 400 MHz): δ = 2.20 (p, *J* = 5.95 Hz, 2H, OCH_2_C*H*_2_CH_2_O), 2.93 (s, 3H, O-SO_2_-C*H*_3_), 4.08 (t, *J* = 5.86 Hz, 2H, OCH_2_ CH_2_C*H*_2_O), 4.41 (t, *J* = 6.07 Hz, 2H, OC*H*_2_CH_2_CH_2_O), 7.00 (d, *J* = 8.95 Hz, 1H, Benzthia-5-*H*), 7.17 (d, ^4^*J* = 2.39 Hz, 1H, Benzthia-7-*H*), 7.76 (d, *J* = 8.95 Hz, 1H, Benzthia-4-*H*); ESI-MS (m/z): 321.8 (M-H^+^, 100), 323.8 (M-H^+^, 43), 324.8 (M-H^+^, 6).

##### 4-(2-chlorobenzothiazol-6-yloxy)butyl methanesulfonate (31e)

**30c** (0.5 g, 1.95 mmol) solved in 20 ml dichlormethane were added to triethylamine (0.3 g, 2.9 mmol). The solution was cooled under argon atmosphere to −10°C. Methanesulfonic chloride (0.27 g, 2.3 mmol) dissolved in 10 ml dichlormethane was added dropwise. The solution was stirred 15 min by −10°C and 45 min by room temperature, poured into ice water and twice extracted with a sodium carbonate solution. The organic layer was dried over sodium sulfate and evaporated to give a yellow oil (0.54 g, yield 82%); ^1^H-NMR (CDCl_3_, 400 MHz): δ = 1.91–1.93 (m, 4H, OCH_2_C*H*_2_C*H*_2_CH_2_O), 2.95 (s, 3H, O-SO_2_-C*H*_3_),3.99 (t, *J* = 5.61 Hz, 2H, OCH_2_CH_2_CH_2_C*H*_2_O), 4.04 (t, *J* = 5.86 Hz, 2H, OC*H*_2_CH_2_CH_2_CH_2_O), 6.99 (d, *J* = 8.95 Hz, 1H, Benzthia-5-*H*), 7.15 (d, ^4^*J* = 2.46 Hz, 1H, Benzthia-7-*H*), 7.75 (d, *J* = 8.95 Hz, 1H, Benzthia-4-*H*); ESI-MS (m/z): 335.8 (M-H^+^, 100), 336.8 (M-H^+^, 15), 337.7 (M-H^+^, 42).

##### 6-(2-(4-phenylpiperazin-1-yl)ethoxy)benzothiazole hydrogenoxalate (13)

(0.6 mmol) was dissolved in acetone. *N*-phenylpiperazine (0.09 g, 0.6 mmol), anhydrous potassium carbonate (0.17 g, 1.2 mmol) and catalytic amounts of potassium iodide were added. The suspension was refluxed for 48 h, filtered and the yellow filtrate was evaporated to dry. The resulting yellowish raw product was purified by column chromatography. The resulting colorless oil was precipitated with anhydrous oxalic acid and dried to give a white solid (0.16 g, yield: 31%); mp 215°C (decomp.); C_19_H_21_N_3_OS × C_2_H_2_${\text{O}}_{4;}^{1}$H-NMR (d_6_ DMSO, 300 MHz) δ = 3.20 (m, 4H, Pip-2-*H*, -6-*H*), 3.36–3.39 (m, 6H, OCH_2_C*H*_2_, Pip-3-*H*, -5-*H*), 4.45 (m, 2H, OC*H*_2_CH_2_), 6.90 (t, *J* = 7.23 Hz, 1H, Ar-4-*H*), 7.06 (d, *J* = 8.04 Hz, 2H, Ar-2-*H*, -6-*H*), 7.26–7.34 (m, 3H, Benzthia-5-*H*, Ar-3-*H*, -5-*H*), 7.87 (d, ^4^*J* = 1.52 Hz, 1H, Benzthia-*7*-*H*), 8.08 (d, *J* = 8.83 Hz, 1H, Benzthia-4-*H*), 9.29 (s, 1H, Benzthia-2-*H*); ^13^C-NMR (d_6_ DMSO, 75 MHz): δ = 45.83 (Pip-3-*C*, -5-*C*), 51.58 (Pip-2-*C*, -6-*C*), 54.73 (OCH_2_*C*H_2_), 63.45 (O*C*H_2_CH_2_), 106.01 (Benzthia-7-*C*), 115.79 (Ar-*C*-2,-*C*-6), 116.06 (Benzthia-5-*C*), 119.74 (Ar-*C*-4), 123.52 (Benzthia-4-*C*), 129.05 (Ar-*C*-3, -*C*-5), 134.95 (Benzthia-7a-*C*), 147.94 (Benzthia-3a-*C*), 149.77 (Ar-*C*-1), 153.82 (Benzthia-2-*C*), 155.81 (Benzthia-6-*C*), 162.14 ((*C*OOH)_2_); ESI-MS (m/z): 340.1 (M-H^+^, 100), 341.1 (M-H^+^, 22), 342.1 (M-H^+^, 7). CHN calc.: C 57.52, H 5.52, N 9.58; CHN found: C 57.69, H 5.38, N 9.67.

##### 2-chloro-6-(2-(4-phenylpiperazin-1-yl)ethoxy)benzothiazole dihydrogenoxalate (14)

**31b** (0.6 mmol) was dissolved in acetone. *N*-phenylpiperazine (0.09 g, 0.6 mmol), anhydrous potassium carbonate (0.17 g, 1.2 mmol) and catalytic amounts of potassium iodide were added. The suspension was refluxed for 48 h, filtered and the yellow filtrate was evaporated to dry. The resulting yellowish raw product was purified by column chromatography. The resulting colorless oil was precipitated with anhydrous oxalic acid and dried to give a white solid (yield: 41%); mp 168°C (decomp.); C_19_H_20_ClN_3_OS × 2 C_2_H_2_O_4_;^1^H-NMR (CDCl_3_, 400 MHz): δ = 2.68–2.70 (m, 4H, Pip-2-*H*, -6-*H*), 2.84 (t, *J* = 5.70 Hz, 2H, OCH_2_C*H*_2_), 3.15–3.17 (m, 4H, Pip-3-*H*, -5-*H*), 4.13 (t, *J* = 5.70 Hz, 2H, OC*H*_2_CH_2_), 6.79 (t, *J* = 7.29 Hz, 1H, Ar-4-*H*), 6.86 (d, *J* = 7.91 Hz, 2H, Ar-2-*H*, -6-*H*), 7.03 (d, *J* = 8.96, 1H, Benzthia-5-*H*), 7.18–7.20 (m, 2H, Ar-3-*H*, -5-*H*), 7.21 (d, ^4^*J* = 1.93 Hz, 1H, Benzthia-*7*-*H*), 7.75 (d, *J* = 8.96 Hz, 1H, Benzthia-4-*H*); ^13^C-NMR (d_6_ DMSO, 100 MHz): δ = 46.15 (Pip-3-*C*, -5-*C*), 51.76 (Pip-2-*C*, -6-*C*), 54.89 (OCH_2_*C*H_2_), 63.82(O*C*H_2_CH_2_), 106.24 (Benzthia-7-*C*), 115.74 (Ar-*C*-2,-*C*-6), 116.46 (Benzthia-5-*C*), 119.63 (Ar-*C*-4), 123.10 (Benzthia-4-*C*), 129.04 (Ar-*C*-3, -*C*-5), 137.03 (Benzthia-7a-*C*), 145.05 (Benzthia-3a-*C*), 149.86 (Ar-*C*-1), 149.92 (Benzthia-2-*C*), 156.13 (Benzthia-6-*C*), 162.18 ((*C*OOH)_2_); ESI-MS (m/z): 374.1 (M-H^+^, 100), 376.1 (M-H^+^, 38), 377.1 (M-H^+^, 8). CHN cacl.: C 49.87, H 4.37, N 7.59; CHN found: C 49.67, H 4.38, N 7.51.

##### 6-(3-(4-phenylpiperazin-1-yl)propoxy)benzothiazole hydrogenoxalate (16)

**31c** (0.6 mmol) was dissolved in acetone. *N*-phenylpiperazine (0.09 g, 0.6 mmol), anhydrous potassium carbonate (0.17 g, 1.2 mmol) and catalytic amounts of potassium iodide were added. The suspension was refluxed for 48 h, filtered and the yellow filtrate was evaporated to dry. The resulting yellowish raw product was purified by column chromatography. The resulting colorless oil was precipitated with anhydrous oxalic acid and dried to give a white solid (yield: 79%); mp 208–211°C (decomp.); C_20_H_23_N_3_OS × 1.5 C_2_H_2_O_4_ × 0.25 H_2_O; ^1^H-NMR (d_6_ DMSO, 300 MHz): δ = 2.17–2.22 (m, 2H, OCH_2_C*H*_2_CH_2_), 3.21–3.30 (m, 6H, Pip-2-*H*, -6-*H*, OCH_2_ CH_2_C*H*_2_), 3.40 (m, 4H, Pip-3-*H*, -5-*H*), 4.15 (t; *J* = 5.95 Hz, 2H, OC*H*_2_CH_2_CH_2_), 6.85 (t, *J* = 7.25 Hz, 1H, Ar-4-*H*), 7.00 (d, *J* = 7.99 Hz, 2H, Ar-2-*H*, -6-*H*), 7.15 (d, *J* = 8.93 Hz, 1H, Benzthia-5-*H*), 7.26 (t, *J* = 7.93 Hz, 2H, Ar-3-*H*, -5-*H*), 7.73 (d, ^4^*J* = 2.44 Hz, 1H, Benzthia-7-*H*), 7.98 (d, *J* = 8.93 Hz, 1H, Benzthia-4-*H*), 9.19 (s, 1H, Benzthia-2-*H*); ^13^C-NMR (d_6_ DMSO, 75 MHz): δ = 23.66 (OCH_2_*C*H_2_CH_2_), 45.80 (Pip-3-*C*, -5-*C*), 51.00 (Pip-2-*C*, -6-*C*), 53.15 (OCH_2_CH_2_*C*H_2_), 65.68 (O*C*H_2_CH_2_CH_2_), 105.60 (Benzthia-7-*C*), 115.83 (Ar-2-*C*, -6-*C*), 115.98 (Benzthia-5-*C*), 119.81 (Ar-4-*C*), 123.44 (Benzthia-4-*C*), 129.06 (Ar-3-*C*, -5-*C*), 134.98 (Benzthia-7a-*C*), 147.64 (Benzthia-3a-*C*), 149.71 (Ar-1-*C*), 153.33 (Benzthia-2-*C*), 156.42 (Benzthia-6-*C*), 163.24 ((*C*OOH)_2_); ESI-MS (m/z): 354.0 (M-H^+^, 100), 355.0 (M-H^+^, 24), 356.0 (M-H^+^, 7). CHN cacl.: C 56.03, H 5.42, N 8.52; CHN found: C 56.12, H 5.29, N 8.40.

##### 2-chloro-6-(3-(4-phenylpiperazin-1-yl) propoxy)benzothiazole dihydrogenoxalate (17)

**31d** (0.6 mmol) was dissolved in acetone. *N*-phenylpiperazine (0.09 g, 0.6 mmol), anhydrous potassium carbonate (0.17 g, 1.2 mmol) and catalytic amounts of potassium iodide were added. The suspension was refluxed for 48 h, filtered and the yellow filtrate was evaporated to dry. The resulting yellowish raw product was purified by column chromatography. The resulting colorless oil was precipitated with anhydrous oxalic acid and dried to give a white solid (0.16 g, yield: 79%); mp 159–161°C (decomp.); C_20_H_22_ClN_3_OS × 2 C2H2O4;^1^H-NMR (d_6_ DMSO, 300 MHz): δ = 2.17–2.24 (m, 2H, OCH_2_C*H*_2_CH_2_), 3.22–3.31 (m, 6H, Pip-2-*H*, -6-*H*, OCH_2_ CH_2_C*H*_2_), 3.40 (m, 4H, Pip-3-*H*, -5-*H*), 4.13 (t; *J* = 5.89 Hz, 2H, OC*H*_2_CH_2_CH_2_), 6.85 (t, *J* = 7.25 Hz, 1H, Ar-4-*H*), 7.00 (d, *J* = 7.99 Hz, 2H, Ar-2-*H*, -6-*H*), 7.16 (d, *J* = 8.96 Hz, 1H, Benzthia-5-*H*), 7.26 (t, *J* = 7.92 Hz, 2H, Ar-3-*H*, -5-*H*), 7.70 (d, ^4^*J* = 2.50 Hz, 1H, Benzthia-7-*H*), 7.87 (d, *J* = 8.96 Hz, 1H, Benzthia-4-*H*); ^13^C-NMR (d_6_ DMSO, 75 MHz) δ = 23.57 (OCH_2_*C*H_2_CH_2_), 45.76 (Pip-3-*C*, -5-*C*), 50.97 (Pip-2-*C*, -6-*C*), 53.07 (OCH_2_CH_2_*C*H_2_), 65.72 (O*C*H_2_CH_2_CH_2_), 105.92 (Benzthia-7-*C*), 115.84 (Ar-2-*C*, -6-*C*), 116.36 (Benzthia-5-*C*), 119.82 (Ar-4-*C*), 123.04 (Benzthia-4-*C*), 129.06 (Ar-3-*C*, -5-*C*), 137.08 (Benzthia-7a-*C*), 144.81 (Benzthia-3a-*C*), 149.60 (Ar-1-*C*), 149.69 (Benzthia-2-*C*), 156.62 (Benzthia-6-*C*), 162.75 ((*C*OOH)_2_); ESI-MS (m/z): 388.2 (M-H^+^, 100), 390.1 (M-H^+^, 38), 391.2 (M-H^+^, 9). CHN calc.: C 50.74, H 4.76, N 7.16; CHN found: C 50.75, H 4.61, N 7.40.

##### 2-chloro-6-(4-(4-(2-methoxyphenyl)piperazin-1-yl) butoxy)benzothiazole hydrogenoxalate (11)

**31e** (0.6 mmol) was dissolved in acetone. 2-methylphenylpiperazine (0.6 mmol), anhydrous potassium carbonate (0.17 g, 1.2 mmol) and catalytic amounts of potassium iodide were added. The suspension was refluxed for 48 h, filtered and the yellow filtrate was evaporated to dry. The resulting yellowish raw product was purified by column chromatography. The resulting colorless oil was precipitated with anhydrous oxalic acid and dried to give a white solid (0.16 g, yield: 34%); mp 126°C; C_22_H_26_ClN_3_O_2_S × 1.5 C_2_H_2_O_4_ × 0.7 H_2_O; ^1^H-NMR (d_6_ DMSO, 300 MHz): δ = 1.76 (m, 4H, OCH_2_C*H*_2_C*H*_2_CH_2_), 3.06–3.23 (m, 10H, OCH_2_CH_2_CH_2_C*H*_2_, Pip-2-*H*, -3-*H*, -5-*H*, -6-*H*), 3.72 (s, 3 H, OC*H*_3_), 4.02 (t, *J* = 5.40 Hz, 2H, OC*H*_2_CH_2_CH_2_CH_2_), 6.83–6.95 (m, 4H, 2-OCH_3_-Ar-3-*H*, -4-*H*, -5-*H*, -6-*H*), 7.10 (d, *J* = 8.95 Hz, 1H, Benzthia-5-*H*), 7.63 (d, ^4^*J* = 2.46 Hz, 1H, Benzthia-7-*H*), 7.80 (d, *J* = 8.95 Hz, 1H, Benzthia-4-*H*);^13^C-NMR (d_6_ DMSO, 75 MHz): δ = 20.37 (OCH_2_CH_2_*C*H_2_CH_2_), 25.78 (OCH_2_*C*H_2_CH_2_CH_2_), 47.20 (Pip-3-*C*, -5-*C*), 51.31 (Pip-2-*C*, -6-*C*), 55.34 (OCH_2_CH_2_CH_2_*C*H_2_), 55.34 (O*C*H_3_), 67.51 (O*C*H_2_CH_2_CH_2_CH_2_), 105.83 (Benzthia-7-*C*), 111.92 (Benzthia-5-*C*), 116.39 (2-OCH_3_-Ar-6-*C*), 118.21 (2-OCH_3_-Ar-3-*C*), 120.81 (2-OCH_3_-Ar-4-*C*), 123.01 (2-OCH_3_-Ar-5-*C*), 123.34 (Benzthia-4-*C*), 137.06 (Benzthia-7a-*C*), 139.51 (Benzthia-3a-*C*), 142.00 (2-OCH_3_-Ar-1-*C*), 144.68 (2-OCH_3_-Ar-2-*C*), 151.83 (Benzthia-2-*C*), 156.86 (Benzthia-6-*C*), 163.21 ((*C*OOH)_2_); ESI-MS (m/z): 432.2 (M-H^+^, 100), 433.6 (M-H^+^, 38), 434.1 (M-H^+^, 39). CHN calc.: C 51.80, H 5.29, N 7.25; CHN found: C 51.91, H 4.99, N 7.38.

##### 2-chloro-6-(4-(4-phenylpiperazin-1-yl)butoxy)benzothiazole dihydrogenoxalate (20)

**31e** (0.16 g, 0.6 mmol) was dissolved in acetone. *N*-phenylpiperazine (0.09 g, 0.6 mmol), anhydrous potassium carbonate (0.17 g, 1.2 mmol) and catalytic amounts of potassium iodide were added. The suspension was refluxed for 48 h, filtered and the yellow filtrate was evaporated to dry. The resulting yellowish raw product was purified by column chromatography. The resulting colorless oil was precipitated with anhydrous oxalic acid and dried to give a white solid (0.16 g, yield: 46%); mp 155–157°C (decomp.); C_21_H_24_ClN_3_OS × 2 C_2_H_2_O_4_;^1^H-NMR (d_6_ DMSO, 300 MHz): δ = 1.82 (m, 4H, OCH_2_C*H*_2_C*H*_2_CH_2_), 3.12–3.39 (m, 10H, OCH_2_CH_2_CH_2_C*H*_2_, Pip-2-*H*, -3-*H*, -5-*H*,-6-*H*), 4.08 (t, *J* = 5.55 Hz, 2H, OC*H*_2_CH_2_CH_2_CH_2_), 6.85 (t, *J* = 7.27 Hz, 1H, Ar-4-*H*), 6.99 (d, *J* = 7.93 Hz, 2H, Ar-2-*H*, -6-*H*), 7.15 (d, *J* = 8.97 Hz, 1H, Benzthia-5-*H*), 7.22 -7.28 (m, 2H, Ar-3-*H*, -5-*H*), 7.68 (d, ^4^*J* = 2.55 Hz, 1H, Benzthia-7-*H*), 7.86 (d, *J* = 8.94 Hz, 1H, Benzthia-4-*H*); ^13^C-NMR (d_6_ DMSO, 75 MHz): δ = 20.34 (OCH_2_CH_2_*C*H_2_CH_2_), 25.78 (OCH_2_*C*H_2_CH_2_CH_2_), 45.66 (Pip-3-*C*, -5-*C*), 50.80 (Pip-2-*C*, -6-*C*), 55.26 (OCH_2_CH_2_CH_2_*C*H_2_), 67.50 (O*C*H_2_CH_2_CH_2_CH_2_), 105.82 (Benzthia-7-*C*), 115.85 (Ar-2-*C*, -6-*C*), 116.38 (Benzthia-5-*C*), 119.85 (Ar-4-*C*), 123.01 (Benzthia-4-*C*), 129.06 (Ar-3-*C*, -5-*C*), 137.07 (Benzthia-7a-*C*), 144.68 (Benzthia-3a-*C*), 149.43 (Benzthia-2-*C*), 149.66 (Ar-1-*C*), 156.85 (Benzthia-6-*C*), 162.79 ((*C*OOH)_2_). ESI-MS (m/z): 402.0 (M-H^+^, 100), 403.8 (M-H^+^, 30), 404.3 (M-H^+^, 27). CHN cacl.: C 51.59, H 4.85, N 7.22; CHN found: C 51.80, H 4.81, N 7.38.

#### Detailed synthesis of 2-chloro-6-(4-(4-phenylpiperazin-1-yl)butoxy)benzothiazole (20)

##### 1-(4-bromobutoxy)-4-nitrobenzene (22c)

**21** (3.0 g, 22 mmol) was diluted into 100 ml acetone. 1,4-dibromobutane (19.0 g, 88 mmol), anhydrous potassium carbonate (3.0 g, 22 mmol) and a catalytic amount of potassium iodide was added. The suspension was heated under reflux for 24 h. After cooling the yellow-white suspension was filtered. The yellow filtrate was evaporated to give a yellow oily raw product. The product was purified by filtration with petrolether (60–90°C) and dicloromethane over silicagel to obtain a pure yellow oil (5.34 g, yield: 90%); ^1^H-NMR (CDCl_3_, 400 MHz): δ = 1.90–2.05 (m, 4H, OCH_2_C*H*_2_C*H*_2_CH_2_Br) 3.42 (t, *J* = 6.37 Hz, 2H, OCH_2_CH_2_CH_2_C*H*_2_Br), 4.02 (t, *J* = 5.78 Hz, 2H, OC*H*_2_CH_2_CH_2_CH_2_Br), 6.87 (d, *J* = 9.15 Hz, 2H, Ar-2-*H*, -6-*H*), 8.11 (d, *J* = 9.13 Hz, 2H, Ar-3-*H*, -5-*H*); ESI-MS m/z: 297.9 (M-Na^+^, 100), 295.9 (M-Na^+^, 98), 296.7 (M-Na^+^, 10).

##### 1-(4-hydroxybutoxy)-4-nitrobenzene (25c)

**22c** (2.74 g, 1.0 mmol) was dissolved in 200 ml of a mixture acetone: water (7:3 v/v). After addition of the base *N,N*-diisopropylamine (1.1 g, 1.1 mmol) the solution was heated in a Parr apparatus to 130°C for 9 h under continuous steering. After cooling and evaporation of acetone, the brown solution was acidified with hydrochloric acid solution (2 N) and extracted with ethyl acetate. The combined organic layers were dried over sodium sulfate. After evaporation of the solvent the crude product was purified by column chromatography to give a white solid (0.89 g, yield: 45%); ^1^H-NMR (d_6_ DMSO, 300 MHz) δ = 1.51–1.60 (m, 2H, OCH_2_CH_2_C*H*_2_CH_2_OH), 1.73–1.80 (m, 2H, OCH_2_C*H*_2_CH_2_CH_2_OH), 3.42–3.48 (m, 2H, OCH_2_CH_2_CH_2_C*H*_2_OH), 4.13 (t, *J* = 6.55 Hz, 2H, OC*H*_2_CH_2_CH_2_CH_2_OH), 4.44 (t, *J* = 5.14 Hz, 1H, OCH_2_CH_2_CH_2_CH_2_O*H*), 7.12 (d, *J* = 9.32 Hz, 2H, Ar-2-*H*, -6-*H*), 8.19 (d, *J* = 9.32 Hz, 2H, Ar-3-*H*, -5-*H*); ESI-MS (m/z): 211.7 (M-H^+^, 100), 212.9 (M-H^+^, 23), 213.8 (M-H^+^, 13).

##### 4-(4-hydroxybutoxy)-aniline (26c)

**25c** (0.89 g, 4.2 mmol) was dissolved with 50 ml ethyl acetate. One hundred milligram Pd/C (10% m/m) and a few drops glacial acetic acid were added. The suspension was stirred at room temperature in a Parr apparatus with a pressure of 5 bar hydrogen for 1 h. After filtration of the catalyst the yellow-brown solution was evaporated to dryness to give a brownish oil (0.76 g, yield: 100%)0.1H-NMR (d_6_ DMSO, 400 MHz) δ = 1.55–1.63 (m, 2H, OCH_2_CH_2_C*H*_2_CH_2_OH), 1.69–1.76 (m, 2H, OCH_2_C*H*_2_CH_2_CH_2_OH), 3.49 (t, *J* = 6.48 Hz, 2H, OCH_2_CH_2_CH_2_C*H*_2_OH), 3.87 (t, *J* = 6.50 Hz, 2H, OC*H*_2_CH_2_CH_2_CH_2_OH), 6.55 (d, *J* = 8.78 Hz, 2H, Ar-2-*H*, -6-*H*), 6.69 (d, *J* = 8.80 Hz, 2H, Ar-3-*H*, -5-*H*); ESI-MS (m/z): 181.8 (M-H^+^, 100), 182.7 (M-H^+^, 12).

##### 2-amino-6-(4-hydroxybutoxy)benzothiazole (27c)

**26c** (0.76 g, 4.4 mmol) and potassium thiocyanate (1.71 g, 17.6 mmol) were solved in 25 ml glacial acetic acid. The resultant brown solution was cooled on an ice bath. Dropwise bromine (0.71 g, 4.4 mmol) dissolved in 5 ml glacial acetic acid was added, while the temperature was held constant at 4°C. Afterwards the orange suspension was stirred at room temperature overnight. The formed orange solid was filtered of and the yellow filtrate was concentrated in vacuum. The yellow-orange solid was purified by column chromatography to give a white solid (0.96 g, yield: 91%); ^1^H-NMR (d_6_ DMSO, 400 MHz) δ = 1.52–1.59 (m, 2H, OCH_2_CH_2_C*H*_2_CH_2_OH), 1.69–1.76 (m, 2H, OCH_2_C*H*_2_CH_2_CH_2_OH), 3.43–3.45 (m, 2H, OCH_2_CH_2_CH_2_C*H*_2_OH), 3.94 (t, *J* = 6.43 Hz, 2H, OC*H*_2_CH_2_CH_2_CH_2_OH), 6.80 (d, *J* = 8.64 Hz, 1H, Benzthia-5-*H*), 7.19 (s, br, 2H, Benzthia-2-N*H*_2_), 7.21 (d, *J* = 8.95 Hz, 1H, Benzthia-4-*H*), 7.27 (d, ^4^*J* = 2.07 Hz, 1H, Benzthia-7-*H*; ESI-MS (m/z): 238.9 (M-H^+^, 100), 239.9 (M-H^+^, 13), 240.9 (M-H^+^, 6).

##### 2-chloro-6-(4-hydroxybutoxy)benzothiazole (30c)

In a three-neck round bottom flask anhydrous copper(II)chloride (0.65 g, 4.8 mmol) was suspended in 25 ml of dry acetonitrile. *t*-Butylnitrite (0.62 g, 6.0 mmol) was added and stirred at room temperature for 10 min to build a black suspension. **27c** (0.96 g, 4.0 mmol) was dissolved in a mixture of 25 ml dry acetonitrile and 10 ml dry methanol and added dropwise to the black suspension in the flask. The suspension was stirred 1.5 h at room temperature and 2.5 h at 65°C. After the suspension was evaporated with the addition of silicagel to a dry yellow-brown solid, it was purified by column chromatography to yellowish oil (0.5 g, yield: 48%); ^1^H-NMR (d_6_ DMSO, 300 MHz) δ = 1.52–1.61 (m, 2H, OCH_2_CH_2_C*H*_2_CH_2_OH), 1.73–1.80 (m, 2H, OCH_2_C*H*_2_CH_2_CH_2_OH), 3.42–3.48 (m, 2H, OCH_2_CH_2_CH_2_C*H*_2_OH), 4.04 (t, *J* = 6.40 Hz, 2H, OC*H*_2_CH_2_CH_2_CH_2_OH), 4.45 (t, *J* = 4.74 Hz, 1H, OCH_2_CH_2_CH_2_CH_2_O*H*), 7.12 (d, *J* = 8.94 Hz, 1H, Benzthia-5-*H*), 7.66 (d, ^4^*J* = 2.58 Hz, 1H, Benzthia-7-*H*), 7.83 (d, *J* = 8.95 Hz, 1H, Benzthia-4-*H*); ESI-MS (m/z): 257.8 (M-H^+^, 100), 258.9 (M-H^+^, 13), 259.9 (M-H^+^, 39).

##### 4-(2-chlorobenzothiazol-6-yloxy)butyl methanesulfonate (31e)

**30c** (0.5 g, 1.95 mmol) solved in 20 ml dichlormethane were added to triethylamine (0.3 g, 2.9 mmol). The solution was cooled under argon atmosphere to–10°C. Methanesulfonic chloride (0.27 g, 2.3 mmol) dissolved in 10 ml dichlormethane was added dropwise. The solution was stirred 15 min by–10°C and 45 min by room temperature, poured into ice water and twice extracted with a sodium carbonate solution. The organic layer was dried over sodium sulfate and evaporated to give a yellow oil (0.54 g, yield 82%);^1^H-NMR (CDCl_3_, 400 MHz) δ = 1.91–1.93 (m, 4H, OCH_2_C*H*_2_C*H*_2_CH_2_O), 2.95 (s, 3H, O-SO_2_-C*H*_3_),3.99 (t, *J* = 5.61 Hz, 2H, OCH_2_CH_2_CH_2_C*H*_2_O), 4.04 (t, *J* = 5.86 Hz, 2H, OC*H*_2_CH_2_CH_2_CH_2_O), 6.99 (d, *J* = 8.95 Hz, 1H, Benzthia-5-*H*), 7.15 (d, ^4^*J* = 2.46 Hz, 1H, Benzthia-7-*H*), 7.75 (d, *J* = 8.95 Hz, 1H, Benzthia-4-*H*); ESI-MS (m/z): 335.8 (M-H^+^, 100), 336.8 (M-H^+^, 15), 337.7 (M-H^+^, 42).

##### 2-chloro-6-(4-(4-phenylpiperazin-1-yl)butoxy)benzothiazol dihydrogenoxalate (20)

**31e** (0.16 g, 0.6 mmol) was dissolved in acetone. *N*-phenylpiperazine (0.09 g, 0.6 mmol), anhydrous potassium carbonate (0.17 g, 1.2 mmol) and catalytic amounts of potassium iodide were added. The suspension was refluxed for 48 h, filtered and the yellow filtrate was evaporated to dry. The resulting yellowish raw product was purified by column chromatography. The resulting colorless oil was precipitated with anhydrous oxalic acid and dried to give a white solid (0.16 g, yield: 46%); mp 155–157°C (decomp.); ^1^H-NMR (d_6_ DMSO, 300 MHz) δ = 1.82 (m, 4H, OCH_2_C*H*_2_C*H*_2_CH_2_), 3.12–3.39 (m, 10H, OCH_2_CH_2_CH_2_C*H*_2_, Pip-2-*H*, -3-*H*, -5-*H*,-6-*H*), 4.08 (t, *J* = 5.55 Hz, 2H, OC*H*_2_CH_2_CH_2_CH_2_), 6.85 (t, *J* = 7.27 Hz, 1H, Ar-4-*H*), 6.99 (d, *J* = 7.93 Hz, 2H, Ar-2-*H*, -6-*H*), 7.15 (d, *J* = 8.97 Hz, 1H, Benzthia-5-*H*), 7.22 -7.28 (m, 2H, Ar-3-*H*, -5-*H*), 7.68 (d, ^4^*J* = 2.55 Hz, 1H, Benzthia-7-*H*), 7.86 (d, *J* = 8.94 Hz, 1H, Benzthia-4-*H*); ^13^C-NMR (d_6_ DMSO, 75 MHz) δ = 20.34 (OCH_2_CH_2_*C*H_2_CH_2_), 25.78 (OCH_2_*C*H_2_CH_2_CH_2_), 45.66 (Pip-3-*C*, -5-*C*), 50.80 (Pip-2-*C*, -6-*C*), 55.26 (OCH_2_CH_2_CH_2_*C*H_2_), 67.50 (O*C*H_2_CH_2_CH_2_CH_2_), 105.82 (Benzthia-7-*C*), 115.85 (Ar-2-*C*, -6-*C*), 116.38 (Benzthia-5-*C*), 119.85 (Ar-4-*C*), 123.01 (Benzthia-4-*C*), 129.06 (Ar-3-*C*, -5-*C*), 137.07 (Benzthia-7a-*C*), 144.68 (Benzthia-3a-*C*), 149.43 (Benzthia-2-*C*), 149.66 (Ar-1-*C*), 156.85 (Benzthia-6-*C*), 162.79 ((*C*OOH)_2_); ESI-MS (m/z): 402.0 (M-H^+^, 100), 403.8 (M-H^+^, 30), 404.3 (M-H^+^, 27). CHN calc.: C 51.59, H 4.85, N 7.22; CHN found: C 51.80, H 4.81, N 7.38.

### Pharmacological assay descriptions

#### General remarks

Competition binding data were analyzed by Graph-Pad Prism™(2000, version 3.02, San Diego, CA, USA), using nonlinear least squares fit. Affinity values (*K*_i_) were expressed as mean ± standard deviation [nM ± S.DE.M.]. The values for *K*_i_ and p*K*_i_ were calculated for compounds **3-20** are listed in Tables 1, **3**.

**Table 1:** Dopamine *h*D_2S_R and *h*D_3_R binding affinities of ligands **1**, **2** and final products **3-20**.

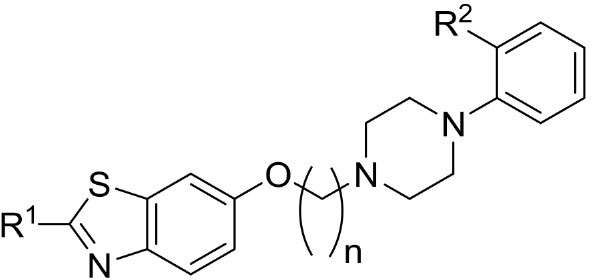					
**Compound**	**R^1^**	**R^2^**	***n***	***h*D_2S_R affinity^a^**	***h*D_3_R affinity^b^**
				***K*_i_ [nM ± SEM]**	***K*_i_ [nM ± SEM]**
1^c^	–	–	–	61	0.92
2^d^	–	–	–	8.7	–
3	H_2_N	OCH_3_	2	26 ± 12	23 ± 2
4	H	OCH_3_	2	35 ± 10	18 ± 3.5
5	Cl	OCH_3_	2	16 ± 8	46 ± 29
6	H_2_N	OCH_3_	3	5.5 ± 0.6	1.7 ± 0.8
7	H	OCH_3_	3	6.8 ± 0.9	8.7 ± 4.8
8	Cl	OCH_3_	3	16 ± 1.1	22 ± 13
9	H_2_N	OCH_3_	4	2.8 ± 0.8	3.0 ± 1.6
10	H	OCH_3_	4	3.2 ± 0.4	8.5 ± 2.2
11	Cl	OCH_3_	4	11 ± 1.9	11 ± 3.9
12	H_2_N	H	2	90 ± 13	304 ± 67
13	H	H	2	439 ± 238	338 ± 192
14	Cl	H	2	540 ± 279	424 ± 230
15	H_2_N	H	3	45 ± 13	41 ± 29
16	H	H	3	134 ± 58	73 ± 37
17	Cl	H	3	127 ± 65	37 ± 18
18	H_2_N	H	4	43 ± 11	16 ± 11
19	H	H	4	85 ± 31	43 ± 22
20	Cl	H	4	138 ± 124	65 ± 17

a,b *Affinity to D_2S_Rs and D_3_Rs determined as described previously (Hayes et al., 1992; Sokoloff et al., 1992; Noeske et al., 2006; Bocker et al., 2007; Boeckler and Gmeiner, 2007)*.

c *Value described in literature (Sukalovic et al., 2005)*.

d *Value described in literature (Tomic et al., 2007)*.

e *Value described in literature (Moller et al., 2014)*.

#### The *in Vitro* affinity

The *in vitro* affinity of the new compounds **3-20** for *h*D_2S_R and *h*D_3_R was determined using radioligand binding studies as described previously (Hayes et al., 1992; Sokoloff et al., 1992; Noeske et al., 2006; Bocker et al., 2007; Boeckler and Gmeiner, 2007). Membrane preparations of CHO-cells stably expressing *h*D_2S_R, the short splicing variant of that receptor subtype, and *h*D_3_R (Hopkins et al., 2004) were used for displacement studies (Sokoloff et al., 1992). In brief, [^3^H]spiperone (0.2 nM) served as radioligand and nonspecific binding was determined in the presence of **1** (10 μM). Stock solutions (10 mM) of test compounds were prepared with pure DMSO. They were diluted in binding puffer (120 mM NaCl, 5 mM KCl, 2 mM CaCl_2_, 1 mM MgCl_2_, 50 mM TRIS/HCl, pH 7.4) to give a final concentration range of 0.01–100,000 nM, and this was depending on the affinity of the test compound. The assay (containing membranes, [^3^H]spiperone and test compounds in a final volume of 0.2 ml) was incubated for 2 h at RT and terminated by rapid filtration through GF/B glass fiber filters (PerkinElmer Life Sciences, Rodgau, Germany) pre-treated with 0.3% polyethylenimine (Sigma–Aldrich, Taufkirchen, Germany) using an Inotech cell harvester (Inotech AG, Dottikon, Switzerland). Unbound radioligand was removed by three washing steps with 0.3 ml/well of ice-cold 50 mM Tris-HCl buffer, pH 7.4, containing 120 mM NaCl. Radioactivity was counted using a 1450 MicroBeta Trilux scintillation counter (PerkinElmer Life Sciences, Rodgau, Germany). To achieve a detailed screening, the compounds have been tested at seven concentrations in triplicates carried out in at least three independent experiments.

### Determination of physicochemical properties

Lipophilicity (clogP), water solubility (clogS), molecular weight (MW), and number of rotatable bonds (NROTB) of Lipinski's rule of five were calculated using Molinspiration online property calculation toolkit, and the computational tool Osiris Property explorer (Table 2; Hopkins et al., 2004; Hou et al., 2007). Drug-likeness model score (a combined outcome of physicochemical properties, pharmacokinetics and pharmacodynamics of a compound and is represented by a numerical value was computed by MolSoft software for the 18 compounds (**3-20**) under study (Table 3; Drug-likeness and molecular property prediction^1^; Tarcsay et al., 2012). Moreover, topological polar surface area (TPSA) was calculated with software package Molinspiration Depiction Software (Table 2; Tarcsay et al., 2012).

**Table 2:** Drug-likeness calculations and Lipinski parameters for final products **3-20**.

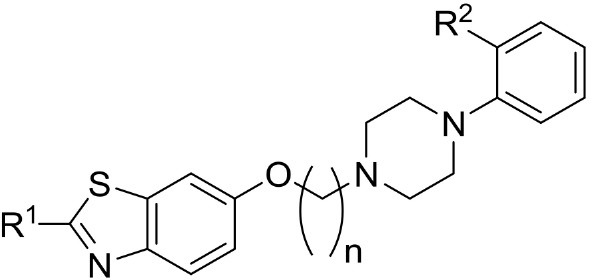							
**Compound**	**MW^a^**	**TPSA^b^**	**NROTB^c^**	**NHBA^d^**	**NHBD^e^**	**cLogS^f^**	**cLogP^g^**
1	417.2	44.8	8	3	1	−5.2	4.5
2	366.2	53.6	7	4	1	−3.9	3.4
3	384.2	63.9	6	5	2	−4.1	3.6
4	369.2	37.8	6	5	0	−3.9	4.0
5	403.1	37.8	6	5	0	−4.7	4.8
6	398.2	63.9	7	5	2	−4.3	3.9
7	383.2	37.8	7	5	0	−4.1	4.3
8	417.1	37.8	7	5	0	−4.8	5.1
9	412.2	63.9	8	5	2	−4.7	4.2
10	397.2	37.8	8	5	0	−4.5	4.6
11	431.1	37.8	8	5	0	−5.2	5.4
12	354.2	54.6	5	4	2	−3.3	3.6
13	339.1	28.6	5	4	0	−3.1	4.0
14	373.1	28.6	5	4	0	−3.8	4.8
15	368.2	54.6	6	4	2	−3.5	3.9
16	353.2	28.6	6	4	0	−3.3	4.3
17	387.1	28.6	6	4	0	−4.0	5.1
18	382.2	54.6	7-8	4	2	−3.9	4.2
19	367.2	28.6	7-6	4	0	−3.7	4.6
20	401.1	28.6	7	4	0	−4.4	5.4

a *Molecular weight*.

b *Topological polar surface area*.

c *Number of rotatable bonds*.

d *Number of hydrogen bonds acceptors*.

e *Number of hydrogen bond donators*.

f *Water solubility (clogS)*.

g *Lipophilicity (clogP)*.

**Table 3:** Antagonist affinities at *h*D_2S_R and *h*D_3_R and values of LE, LELP, and LipE for final products **3-20**.

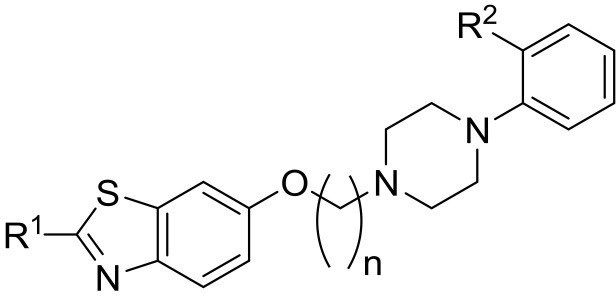									
**Compound**	***h*D_2S_R affinity^a^ p*k*_i_**	**LE^b^ (D_2_R)**	**LELP^c^ (D_2_R)**	**LipE^d^ (D_2_R)**	***h*D_3_R affinity^a^ p*k*_i_**	**LE^b^ (D_3_R)**	**LELP^c^ (D_3_R)**	**LipE^d^ (D_3_R)**	**Drug-likeness model score^e^**
1	7.2	0.3	14.0	2.7	9.0	0.40	11.20	4.56	0.8
2	8.1	0.4	8.2	4.7	–	–	–	–	0.3
3	7.6	0.4	9.3	4.0	7.6	0.4	9.3	4.0	0.8
4	7.5	0.4	10.3	3.4	7.7	0.4	9.8	3.7	0.4
5	7.8	0.4	12.1	3.0	7.3	0.4	13.1	2.5	0.6
6	7.3	0.4	10.9	3.4	8.8	0.4	9.1	4.9	0.8
7	8.2	0.4	10.5	3.9	8.1	0.4	10.5	3.8	0.4
8	7.8	0.4	13.4	2.7	7.7	0.4	13.8	2.6	0.6
9	8.6	0.4	10.5	4.4	8.5	0.4	10.5	4.3	0.7
10	8.5	0.4	10.9	3.9	8.1	0.4	11.7	3.5	0.4
11	9.0	0.4	12.8	3.6	8.0	0.4	14.1	2.6	0.6
12	7.1	0.4	9.3	3.4	6.5	0.4	10.1	2.9	1.0
13	6.4	0.4	11.2	2.4	6.5	0.4	10.9	2.5	0.7
14	6.3	0.3	14.2	1.5	6.4	0.4	13.8	1.6	0.8
15	7.4	0.4	9.8	3.5	7.4	0.4	9.8	3.5	1.0
16	6.9	0.4	11.3	2.6	7.1	0.4	11.0	2.9	0.77
17	6.9	0.4	14.2	1.8	7.4	0.4	13.1	2.3	0.8
18	7.4	0.4	11.3	3.2	7.8	0.4	10.4	3.6	1.0
19	7.1	0.4	12.3	2.5	7.4	0.4	11.7	2.8	0.7
20	6.9	0.4	15.3	1.5	7.2	0.4	14.9	1.8	0.8

a *Affinity calculated as pK_i_ value*.

b *Ligand efficiency*.

c *Lipophilicity-dependent ligand efficiency*.

d *Lipophilic efficiency*.

e *Molinspiration software or free molecular property calculation services (Molinspiration software or free molecular property calculation services [last accessed 2017 April 07])*.

### Metric analyses

The values for LE, LELP, and LipE were calculated for the compounds **3-20** based on their affinities for *h*D_2S_R and *h*D_3_R to further validate their drug-likeness and assessment as prospective new leads (Table 3). These widely used three metrics are substantial models in drug discovery and are simple for evaluating whether a ligand derives its potency from optimal fit with the target protein or simply by virtue of making many contacts (Molinspiration software or free molecular property calculation services [last accessed 2017 April 07]^2^; Kuntz et al., 1999; Meanwell, 2011; Tarcsay et al., 2012; Schreeb et al., 2013; Hopkins et al., 2014; Shultz, 2014). The values for LE, LELP, and LipE were calculated following equations 1, 2, and 3 (Molinspiration software or free molecular property calculation services [last accessed 2017 April 07]; Kuntz et al., 1999; Leeson and Springthorpe, 2007; Hopkins et al., 2014), respectively:

(1) $$\begin{array}{rcl} \text{LE} & = & \left(1.37/\text{HA}\right).\text{p}K_{\text{i}} \end{array}$$

(2) $$\begin{array}{rcl} \text{LELP} & = & \text{clogP}/\text{LE} \end{array}$$

(3) $$\begin{array}{rcl} \text{LipE} & = & \text{p}K_{\text{i}}-\text{clogP} \end{array}$$

## Results

### Chemistry

**SCHEME 1:**
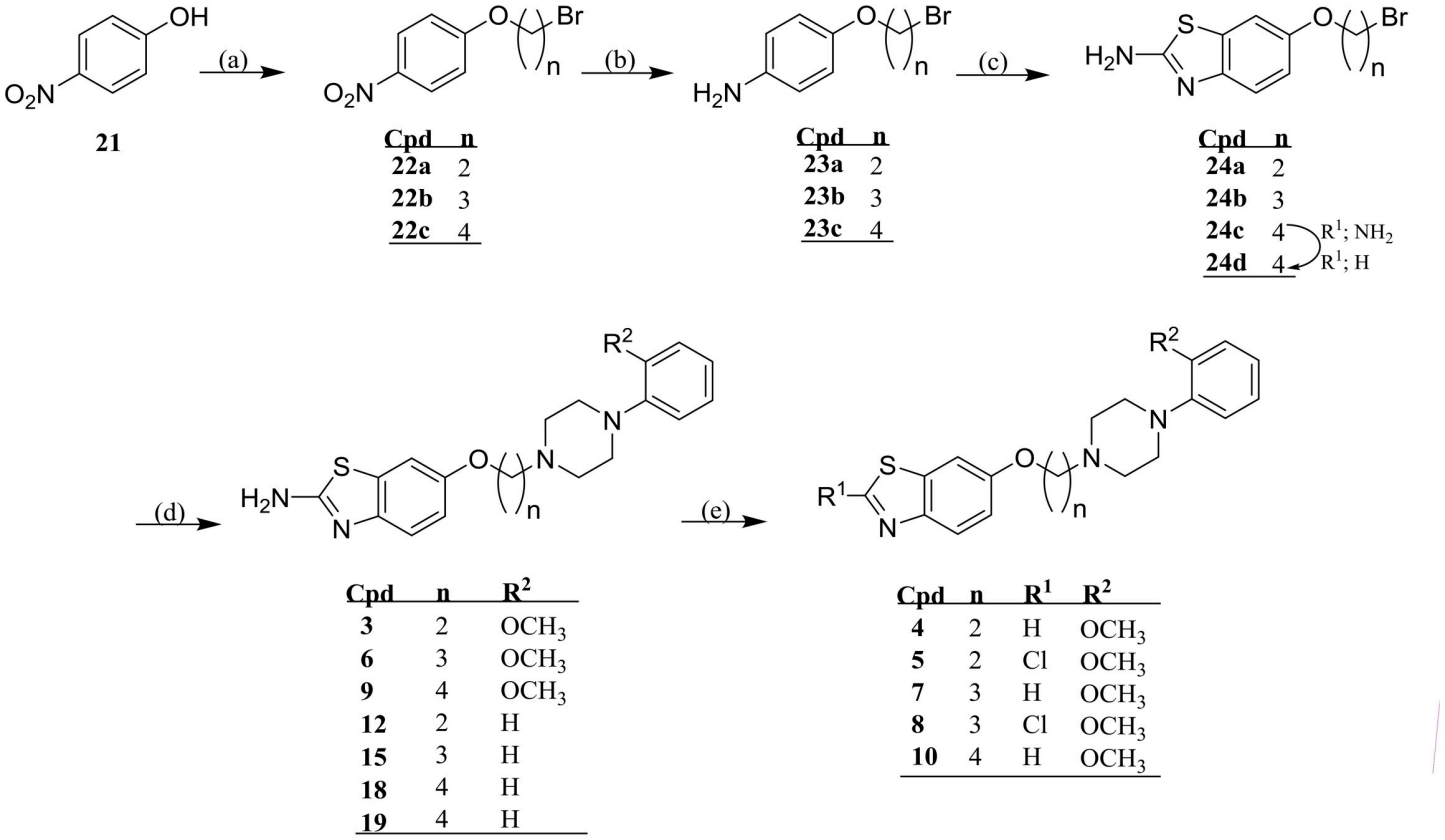
Synthesis of final products **3-10**, **12**, **15**, **18**, and **19**. Reaction conditions: **(a)** Acetone, K_2_CO_3_, Br-(CH_2_)_n_-Br (*n* = 2–4), KI, 24 h reflux; **(b)** Methanol, Pd/C (10% m/m), 5 bar H_2_, 2 h, RT; **(c)** Acetic acid, KSCN, bromine, 4°C → RT, overnight; **(d)** Acetone, K_2_CO_3_, 1-(2-methoxyphenyl)piperazine (for **1-8**) or *N*-phenylpiperazine (for **10**, **13**, and **16**), KI, 24 h reflux; **(e)** HCl, NaNO_2_, H_3_PO_4_ (50% m/m), −30°C → RT; **(f)** H_3_PO_4_ conc., NaNO_2_, H_3_PO_4_ (50% m/m), −8°C → RT (for **17**).

**SCHEME 2:**
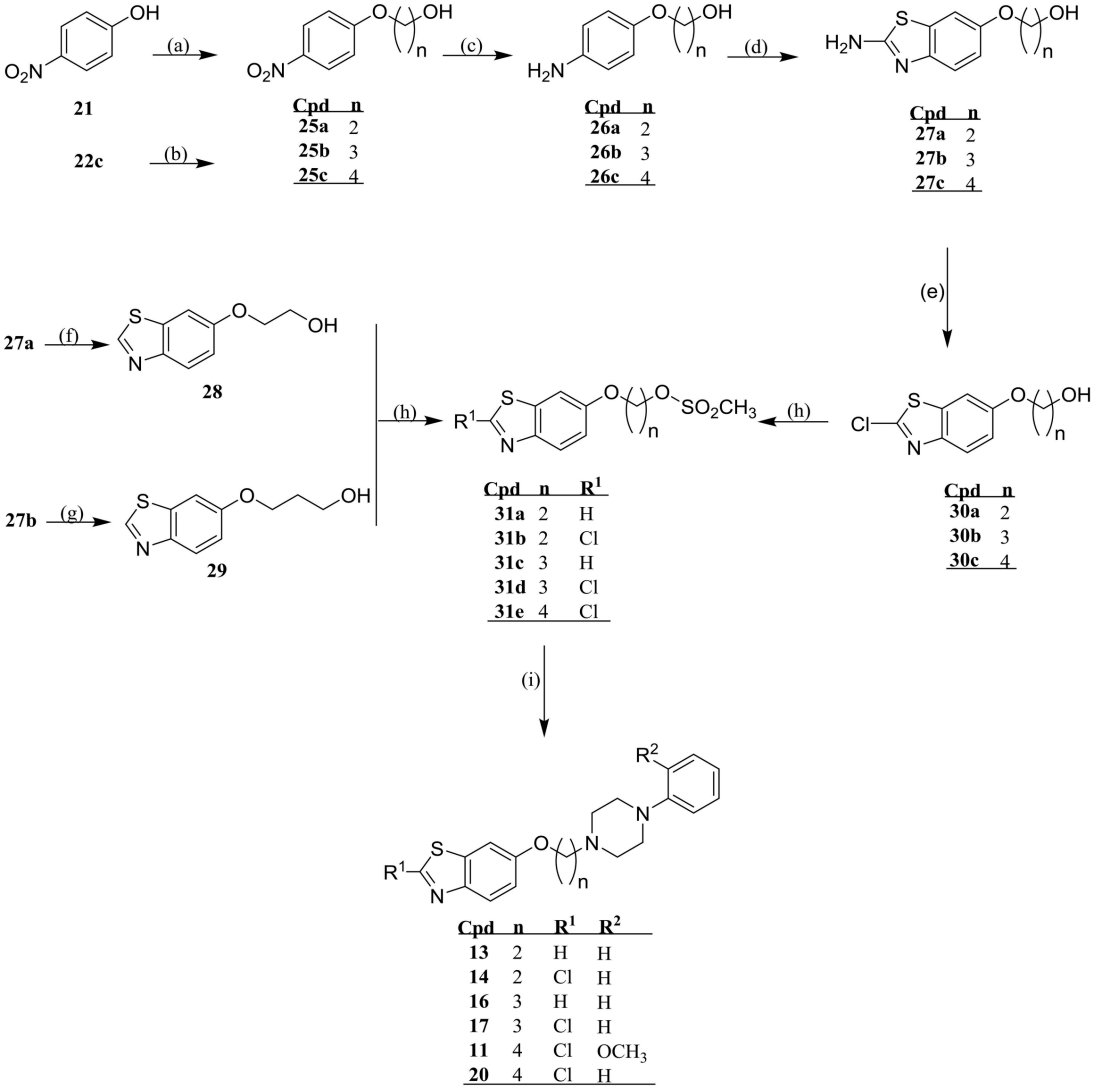
Synthesis of final products **11**, **13**, **14**, **16**, **17**, and **20**. Reaction conditions: **(a)** Acetone, K_2_CO_3_, 2-bromoethanol/3-chloropropan-1-ole, KI, 24 h reflux; **(b)** Acetone:water (7:3 v/v), *N,N*-diisopropylamine, 130°C, 9 h reflux; **(c)** Methanol, Pd/C (10% m/m), 5 bar H_2_ 2 h, RT; **(d)** Acetic acid, KSCN, bromine, 4°C → RT, overnight; **(e)** Acetonitrile, Cu(II)Cl_2_, *t*-butylnitrite, 1.5 h RT, 2.5 h 65°C; **(f)** H_3_PO_4_ conc., NaNO_2_, H_3_PO_4_ (50% m/m), −8°C → RT; **(h)** Dichloromethane, triethylamine, methanesulfonyl chloride, −10°C → RT, 15–45 min; **(i)** Acetone, K_2_CO_3_, 1-(2-methoxyphenyl)piperazine (for **9**)/*N*-phenylpiperazine (for **13**, **14**, **16**, **17**, and **20**), KI, 48 h reflux.

Final compounds **3-20** were prepared starting with an *O*-alkylation of suitable dibromoalkanes under Finkelstein conditions resulting in the formation of the key intermediates **22a-c** which subsequently were reduced to their corresponding aromatic amines (**23a-c**), and were cyclized to their respective 2-aminobenzothiazoyl-6-ω-bromoalkyl ethers **24a-c** under Kaufmann conditions and as previously described (Kaufmann and Bückmann, 1941; Sukalovic et al., 2005). The 2-aminobenz[*d*]thiazoyl-6-ω-bromoalkyl ethers (**24a-c**) were *N*-alkylated according to Finkelstein conditions using 2-methoxyphenylpiperazine or *N*-phenylpiperazine to obtain **3**, **6**, and **9** or **12**, **15**, **18**, and **19**, respectively. For preparation of **4**, **7**, and **10** the respective 2-aminobenzothiazol residue was deaminated by diazotization at 2-position using sodium nitrite and subsequent reductive cleavage of diazonium salt by means of hypophosphoric acid, whereas **5** and **7** were achieved by diazotization conducted in the presence of concentrated hydrochloric acid (Scheme 1). Finally, ligands **11**, **13**, **14**, **16**, **17**, and **20** were synthesized starting from **1** which through *O*-alkylation under Finkelstein conditions with 2-bromoethanol, 3-chloropropan-1-ol, and 1,4-dibromobutane afforded **25a-c**, respectively (Scheme 2).

### *In Vitro* pharmacology

The *h*D_2S_R and *h*D_3_R *in vitro* affinities observed for ligands **3-20** were in the nanomolar concentration range with *K*_i_ values of 2.3–450 [nM] and p*K*_i_ values of 6.4–9.0, and *K*_i_ of 1.7–424 [nM] and p*K*_i_ of 6.4–8.8 for *h*D_2S_R and *h*D_3_R, respectively. The obtained *in vitro* antagonist affinities for the ligands **3-20** were summarized in Table 1.

### Physicochemical and drug-likeness properties

Lipophilicity (clogP), water solubility (clogS), molecular weight (MW), and number of rotatable bonds (NROTB) of Lipinski's rule of five were calculated using Molinspiration online property calculation toolkit, and the computational tool Osiris Property explorer. (Molinspiration software or free molecular property calculation services [last accessed 2017 April 07]^2^). The results observed for the different physicochemical properties are summarized in Table 2.

### Metric properties

The obtained values for LE, LELP, and LipE of all novel ligands **3-20** were measured and are summarized in Table 3.

## Discussion

To evaluate the impact of benzothiazole appendage with the basic 4-phenylpiperazine aryl moiety by an alkyl spacer of varying length on receptor binding, human D_2S_R and D_3_R (*h*D_2S_R and *h*D_3_R) affinities of the described ligands **3-20** were determined using radioligand binding studies on membrane preparations of CHO-cells stably expressing both receptor subtypes. The *h*D_2S_R and *h*D_3_R affinities observed for ligands **3-20** were in the nanomolar concentration range with *K*_i_values of 2.3–450 [nM] and p*K*_i_ values of 6.36–9.0, and *K*_i_ of 1.7–424 [nM] and p*K*_i_ of 6.4–8.8 for *h*D_2S_R and *h*D_3_R, respectively (Tables 1–3). Notably, the results observed in the present study showed that the binding affinities of ligands bearing 2-methoxyphenylpiperazine moiety (**3-11)** showed significantly higher affinities to *h*D_2S_R and *h*D_3_R when compared to their corresponding ligands with an un-substituted 4-phenylpiperazine (**12-20**). Herein, an approximately 34-fold increase in affinity for the 2-methoxy substituted ligands [*K*_i_ (*h*D_2S_R) = 16 ± 8 [nM] for **5**, *K*_i_ (*h*D_2S_R) = 540 ± 279 [nM] for **14**] was observed on *h*D_2S_R, and up to 9-fold higher affinity [*K*_i_(*h*D_3_R) = 46 ± 29 [nM] for **5** vs. *K*_*i*_ (*h*D_3_R) = 424 ± 230 [nM] for **15]** toward the dopamine *h*D_3_R. Interestingly, these results are in agreement with previous findings that showed the strengthening effect of the 2-methoxy substitution on the binding affinity; however, without affecting selectivity profile among both receptor subtypes (Hackling et al., 2003; Boeckler and Gmeiner, 2007; Moller et al., 2014). In addition, our major findings revealed that receptor affinity observed for ligands **3-20** was roughly dependent on the length of the alkyl spacer. Accordingly, the obtained results were expressed in the order of decreasing affinity for tetramethylene ≥ trimethylene > ethylene spacer. In this regard, the ligands having trimethylene spacer showed an up to 5-times higher affinity at *h*D_2S_Rs, and an up to 13-fold higher affinity at *h*D_3_Rs than their respective ethylene spacer analogs [*K*_i_ (*h*D2SR) = 26 ± 12 [nM] and *K*_i_(*h*D3R) = 23 ± 2 [nM] for **3** vs. *K*_i_(*h*D2SR) = 5.5 ± 0.6 [nM] and *K*_i_(*h*D3R) = 1.7 ± 0.8 [nM] for **6**]. However, ligands with tetramethylene spacer exhibited an up to 2-fold higher affinity at *h*D_2S_Rs than their respective trimethylene spacer analogs, and also, with comparable affinity toward *h*D_3_Rs [*K*_i_(*h*D2SR) = 6.8 ± 0.9 [nM] and *K*_i_(*h*D3R) = 8.7 ± 4.8 [nM] for **7** vs. *K*_i_ (*h*D2SR) = 3.2 ± 0.4 [nM] and *K*_i_(*h*D3R) = 8.5 ± 2.2 [nM] for **10**]. Moreover, the flexible substitution pattern at the 2-position of the benzothiazole heterocycle had different effects on *h*D_2S_R and *h*D_3_R affinities. As a result, ligands of ethyl spacer series having a 2-methoxyphenylpiperazin moiety (**3-5**) showed no significant difference in their affinity toward *h*D_2S_Rs and *h*D_3_Rs, whereas ligands of trimethylene and tetramethylene spacer (**6-11**) displayed an affinity toward the *h*D_2S_R in the order of H_2_N- = H- > Cl- with an up to 3-fold [*K*_i_(*h*D2SR) = 5.5 ± 0.6 [nM] and *K*_i_(*h*D3R) = 1.7 ± 0.8 [nM] for **6**] vs. [*K*_i_(*h*D2SR) = 16 ± 1.1 [nM] and *K*_i_(*h*D3R) = 22 ± 13 [nM] for **8**], and 4-fold [*K*_i_(*h*D2SR) = 2.8 ± 0.8 [nM] and *K*_i_(*h*D3R) = 3.0 ± 1.6 [nM] for **7**] vs. [*K*_i_(*h*D2SR) = 11 ± 1.9 [nM] and *K*_i_(*h*D3R) = 11 ± 3.9 [nM] for **11**] increase observed for the 2-amino or the deaminated ligands (**6**, **7**, **9**, and **10**) when compared to their corresponding 2-chloro-analouges (**8** and **11**). Interestingly, the ratio values (*h*D_2S_R/*h*D_3_R) observed for the novel ligands **3-20** were found in the range of 0.3–3, indicating that when compared to the reference compound **1**, which displays a 66-fold higher affinity toward *h*D_3_Rs, ligands **3-20** showed improved dual affinity toward *h*D_2S_Rs and *h*D_3_Rs.

**Figure 2:**
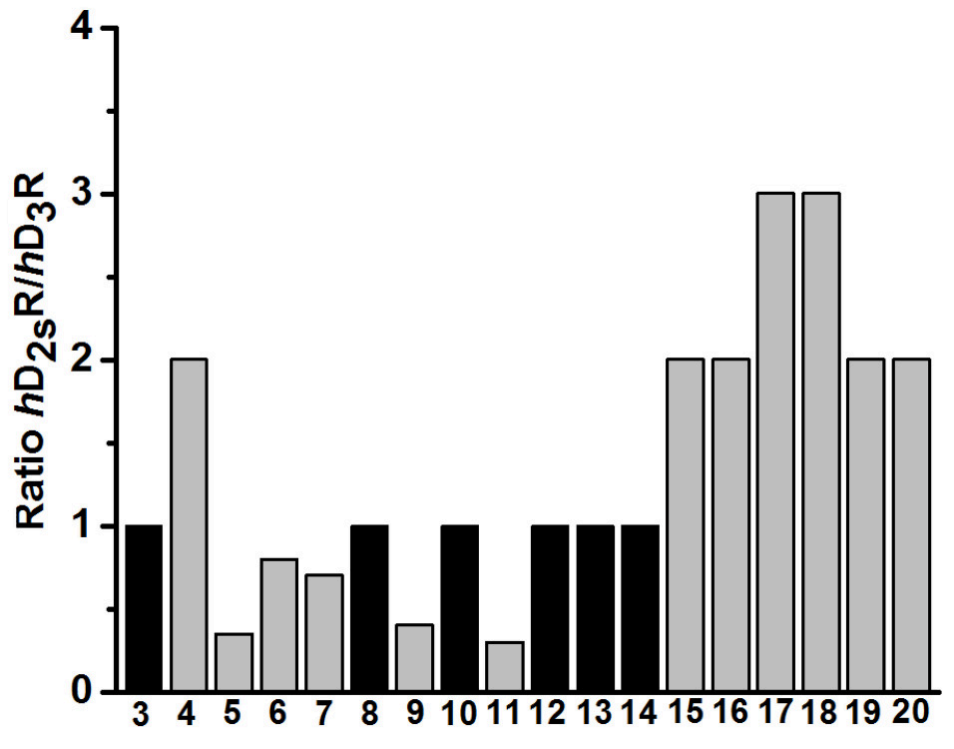
Dopamine *h*D_2S_R/*h*D_3_R binding affinity ratios of final products **3-20**.

Furthermore, the results showed obviously that ligands bearing an unsubstituted (**12-14**) or 4-methoxy-substituted 4-phenylpiperazine moiety (**3**, **8**, and **10**) exhibited in general no significant differences in their binding affinities toward both receptor subtypes (Table 1, Figure 2). Moreover, the affinities to *h*D_2S_Rs observed for ligands **12-20** were found in a high nanomolar range (43–540 [nM]) when compared with ligands **3-11** with binding affinities in the nanomolar range (2.8–26 [nM]) (Table 1). Interestingly, ligand having a tetramethylene spacer (**18**) showed significantly higher affinity to *h*D_3_Rs than its correspondingly 2-chloro-substituted analog **20**, however, in a low nanomolar concentration range with *K*_i_(*h*D_3_R) values of 16 ± 11 [nM] and 65 ± 17 [nM] for **18** and **20**, respectively. The obtained results show that among the current series the highest affinities toward *h*D_2S_Rs and *h*D_3_Rs were achieved with ligands bearing 2-methoxyphenylpiperazinpiperazine moiety connected by a tri- or tetramethylene alkyl spacer with benzothiazole heterocycle which bears an amine group or hydrogen at its 2-position. These structural characteristics were realized and are represented in ligands **6, 7, 9** and **10** with observed affinities in the low nanomolar range toward *h*D_2S_Rs and *h*D_3_Rs.

To assist further the drug discovery/development process and to guide the optimization from a lead compound to a successful drug candidate, various physicochemical parameters have been shown to be useful tools to aid in selecting oral drug candidates (Hopkins et al., 2004; Meanwell, 2011). Therefore, lipophilicity (clogP), water-solubility (clogS), molecular weight (MW), number of rotatable bonds (NROTB), number of hydrogen bond acceptors (NHBA), number of hydrogen bond donators (NHBD), topological polar surface area (TPSA), and drug-likeness score of Lipinski's rule of five were calculated for the current series of ligands (**3-20**) using Molinspiration online property calculation toolkit and Osiris Property explorer (Table 2; Meanwell, 2011; Tarcsay et al., 2012). The clogS value demonstrates the solubility of a drug candidate, which impacts its absorption and distribution properties.(Molinspiration software or free molecular property calculation services [last accessed 2017 April 07]^2^) Accordingly, clogS can be a useful parameter to estimate the ADME properties of a ligand (Hopkins et al., 2014) Solubility of ligands **4**, **12-16**, **18**, and **19** were found in an acceptable range (< −4 clogS). Also, ligands **4**, **12-16**, **18**, and **19** showed lower clogS values, indicating predictable enhanced water solubility, when compared with the reference ligand **1** (Table 2). Moreover, the models of lipophilicity-related physicochemical analyses predictively quantifying target-oriented drug-likeness have for many years been recognized as useful tools in the lead optimization process. This is not unexpected since the influence of clogP in modifying drug potency, pharmacokinetics and toxicity has been established for many years (Meanwell, 2011). Accordingly, it has been proposed that ligands with a clogP < 5 have a more promising drug-likeness profile (Schreeb et al., 2013; Shultz, 2014). Among the current series of ligands, the clogP values, except that for **8**, **11**, **17**, and **20** were found < 5 suggesting their suitability for oral route of administration (Table 2). To promote the assessment of the drug discovery/development process from a lead ligand to a successful drug candidate, other physicochemical parameters have been publicized to be valuable tools to aid in selecting oral drug candidates (Meanwell, 2011). Consequently, it has earlier been suggested that ligands with TPSA values >60 Å^2^ are usually considered as poorly membrane-permeable substances with predictable reduced CNS bioavailability (Hopkins et al., 2004; Meanwell, 2011). Among the synthesized series, the observed TPSA values for compounds **3-20** were in the range of 28–63 Å^2^, imposing more structural optimization of the current class with the aim to generate newer derivatives with improved predictive physicochemical parameters, especially regarding TPSA (Table 2).

To further assist the drug discovery/development process, generally values of LE and LELP being >0.3 and < 7.5, respectively, are validated as promising candidates with drug-likeness (Kuntz et al., 1999). Interestingly, all ligands (**3-20**) showed LE >0.3 for *h*D_2S_Rs and *h*D_3_Rs, whereas the correlation of observed LELP values (*h*D_2S_Rs and *h*D_3_Rs) were not existing, and were found >9 (Table 3). Importantly, the LipE values which are considered to be independent on molecular size of the respective ligand were found for all ligands <5 by considering the affinity of each respective ligand for *h*D_2S_Rs and *h*D_3_Rs. The LipE results observed for the current series further corroborate their drug-likeness (Table 3).

Moreover, drug-likeness model score (a combined outcome of physicochemical properties, pharmacokinetics and pharmacodynamics of a compound and is represented by a numerical value) was computed as previously described by MolSoft software for the ligands **3-20** and summarized in Table 3 (Drug-likeness and molecular property prediction) (Hopkins et al., 2004). It has been proposed that ligands having zero or negative value should not be considered as drug-like candidate. In regard to the developed series **3-20**, except **11** all ligands possessed drug-likeness scores in the range of 0.4–1.00, and with ligands **12, 15**, and **18** showing maximum-likeness scores of 1.0 (Table 3; Figure 2). The results of pharmacological screening and calculated metric parameters are summarized in Tables 1–3.

## Conclusion

Several benzothiazole-based ligands targeting *h*D_2S_R and *h*D_3_R were synthesized by varying the length of alkyl spacer and the substitution at 2-position of benzothiazole and/or 4-phenylpiperazine. Most of these derivatives showed affinities to the *h*D_2S_R and *h*D_3_R in nanomolar concentration range comparable to that of the reference ligands **1** and **2**. The high affinities of the new ligands, especially **9** and **10**, at the *h*D_2S_R and *h*D_3_R confirm the proposed affinity-strengthening effect of benzothiazole heterocylcle toward *h*D2sRs and *h*D_3_Rs. A calculation of physicochemical properties may provide a good assessment to an enhanced drug-likeness of most of the ligands. Especially ligands **9** and **10** with high dual affinities at *h*D_2S_Rs and *h*D_3_Rs [p*K*_i_ (*h*D_2S_R) = 8.6 and p*K*i (*h*D_3_R) = 8.5 for **9**; p*K*_i_ (*h*D_2S_R) = 8.5 and p*K*i (*h*D_3_R) = 8.1 for **10**] show reduced lipophilicity as compared to those calculated properties of **1** and **2**. Combining these results, these two ligands revealed promising LE, LipE, and drug-likeness values, and showed that ligand affinity and selectivity between *h*D_2S_R and *h*D_3_R activation were strongly influenced by the appendage of benzothiazole, as a lipophilic bioisosterical replacement of aryl moiety, the length of alkyl spacer, and the nature of functional groups attached to the benzothiazole and the basic 4-phenylpiperazine moiety.

## Author contributions

BS and HS were responsible for the study concept, design, acquisition and analysis of data. MS, TK, and LW were responsible for the generation, synthesis and pharmacological *in vitro* characterization the novel ligands. MS and BS drafted the manuscript. HS provided critical revision for the manuscript. All authors critically reviewed content and approved final version for publication.

### Conflict of interest statement

The authors declare that the research was conducted in the absence of any commercial or financial relationships that could be construed as a potential conflict of interest.

## Footnotes

^1^Drug-likeness and molecular property prediction, a.f.h.w.m.c.m.

^2^Molinspiration software or free molecular property calculation services [last accessed 2017 April 07], s. Available online at: URL: www.molinspiration.com/cgi-bin/properties.
